# Functional metagenomic analysis of quorum sensing signaling in a nitrifying community

**DOI:** 10.1038/s41522-021-00250-3

**Published:** 2021-10-28

**Authors:** Chuan Hao Tan, Yee Phan Yeo, Muhammad Hafiz, Noele Kai Jing Ng, Sujatha Subramoni, Shireen Taj, Martin Tay, Xie Chao, Staffan Kjelleberg, Scott A. Rice

**Affiliations:** 1 Singapore Centre for Environmental Life Sciences Engineering, Nanyang Technological University, Singapore, Singapore; 2 School of Biological Sciences, Nanyang Technological University, Singapore, Singapore; 3 ithree Institute, The University of Technology Sydney, Sydney, Australia; 4 Present Address: Kemin Industries (Asia) Pte. Ltd, 12 Senoko Drive, Singapore, 758200 Singapore; 5 Present Address: Waterhub, 82 Toh Guan Road East #C2-14, Singapore, 608576 Singapore

**Keywords:** Metagenomics, Microbiology

## Abstract

Quorum sensing (QS) can function to shape the microbial community interactions, composition, and function. In wastewater treatment systems, acylated homoserine lactone (AHL)-based QS has been correlated with the conversion of floccular biomass into microbial granules, as well as EPS production and the nitrogen removal process. However, the role of QS in such complex communities is still not fully understood, including the QS-proficient taxa and the functional QS genes involved. To address these questions, we performed a metagenomic screen for AHL genes in an activated sludge microbial community from the Ulu Pandan wastewater treatment plant (WWTP) in Singapore followed by functional validation of *luxI* activity using AHL biosensors and LC–MSMS profiling. We identified 13 *luxI* and 30 *luxR* homologs from the activated sludge metagenome. Of those genes, two represented a cognate pair of *luxIR* genes belonging to a *Nitrospira* spp. and those genes were demonstrated to be functionally active. The LuxI homolog synthesized AHLs that were consistent with the dominant AHLs in the activated sludge system. Furthermore, the LuxR homolog was shown to bind to and induce expression of the *luxI* promoter, suggesting this represents an autoinduction feedback system, characteristic of QS circuits. Additionally, a second, active promoter was upstream of a gene encoding a protein with a GGDEF/EAL domain, commonly associated with modulating the intracellular concentration of the secondary messenger, c-di-GMP. Thus, the metagenomic approach used here was demonstrated to effectively identify functional QS genes and suggests that *Nitrospira* spp. maybe QS is active in the activated sludge community.

## Introduction

Quorum sensing (QS) is a bacterial communication system that involves production, secretion, and response to signal molecules known as autoinducers and QS has been shown to regulate many bacterial behaviors^1–3^. QS has been extensively studied in a number of model microorganisms under axenic conditions^3–6^. However, most bacteria predominantly exist as a complex community in their natural habitat^7,8^ where interspecies crosstalk may be common and where signaling process potentially play an important role in community function^9,10^. In wastewater treatment systems, interaction via QS signaling has been shown to serve important functions at the community level. For example, an increased AHL concentration was associated with changes in community composition and was linked to enhanced EPS production and biofilm formation in an activated sludge community^11,12^. In our previous studies, the formation of activated sludge granules was correlated with an increased in AHL concentration^13^ and was accompanied by a shift in microbial community species composition. In particular, this change in community members was associated with a reduction in the organisms encoding quorum quenching functions to be dominated by those that encoded QS systems^14^. In terms of wastewater treatment performance, the ammonia removal efficiency was positively correlated with C4-HSL concentration, while C6- and C8-HSL were positively correlated with nitrite removal efficiency^11,15^. Activated sludge communities are highly diverse, commonly comprised of members from *Proteobacteria, Bacteriodetes,* and *Chloroflexi*^16^ and often, many of those taxa are uncultivable or outgrown by faster-growing heterotrophs^17,18^. Because of these challenges, it is often difficult to directly couple QS organisms with their putative functions within the community.

Traditional approaches for environmental samples have used metagenomic clone libraries to identify QS genes and reporter strains to identify the signals and the approximate concentrations of those signals^19–21^. However, this approach has limitations due to the need to construct and screen the entire metagenomic clone library as well as issues with gene expression in a heterologous host^22,23^. Typically, such screens have identified one to three AHL QS genes from the entire community^19–21^. Metagenome sequencing represents a potentially powerful approach to improve the identification of functional genes in complex communities and to assign those genes to specific taxa. For example, a total of 569 *luxI* sequences were found from the analysis of 68 metagenome samples collected from the Global Ocean Sampling^24^, while another in-silico study identified 31 *luxI* sequences from 14 environmental metagenomes^25^. These results highlight the power of metagenomic approaches in the search for AHL QS genes in complex communities. However, these in-silico analyses were not supported with experimental data to demonstrate that the QS genes identified were indeed functional.

In this study, we performed metagenomic sequencing to examine the QS community in the activated sludge from the Ulu Pandan (UP) Wastewater treatment Plant (WWTP) in Singapore. We aimed to: (i) identify and functionally verify the genes involved in AHL QS signaling and (ii) to resolve the taxonomic origins of the putative *luxI* and *luxR* genes to explore the bacterial groups that may communicate via AHL QS in the activated sludge community. We found a cognate *luxIR* pair belonging to *Nitrospira* spp., a widespread genus of nitrite-oxidizing bacteria (NOB)^26^. We found that the *Nitrospira* LuxR (designated as NspR1) regulates the expression of its cognate *luxI* (designated as *nspI*) gene, a hypothetical GGDEF/EAL-domain containing protein and a hypothetical transcription factor protein. We also demonstrated the binding of NspR1 to the promoter region of *nspI* gene in vitro and show that the DNA-binding activity requires the presence of RNA polymerase.

## Results

### Identification and functional characterization of putative *luxIR* genes in an activated sludge community

We have previously shown that the accumulation of specific AHLs was correlated with the granulation process and EPS production^13^. However, those studies did not determine which species were AHL producers or responders to address what functions might be QS controlled, especially in relation to the function of the community. Therefore, this study has undertaken metagenome and cloning-based approaches to identify QS organisms and associated functions. The sludge microbial community was shotgun sequenced and assembled using Roche 454 and Illumina sequencing. The metagenome constructed was 3.7 Gb in size, consisting of 1,056,488 ORFs from 220,511 metagenome contigs. Putative AHL genes were identified based on the KEGG orthology (KO) assignment and validated independently using Pfam. In total, 13 full-length *luxI* homologs that were co-localized with nine cognate *luxR* homologs were identified from the sludge metagenome. In addition, 21 *luxR* ‘solo’ genes, which lacked a cognate *luxI*^27^ were also identified.

**Fig. 1:**
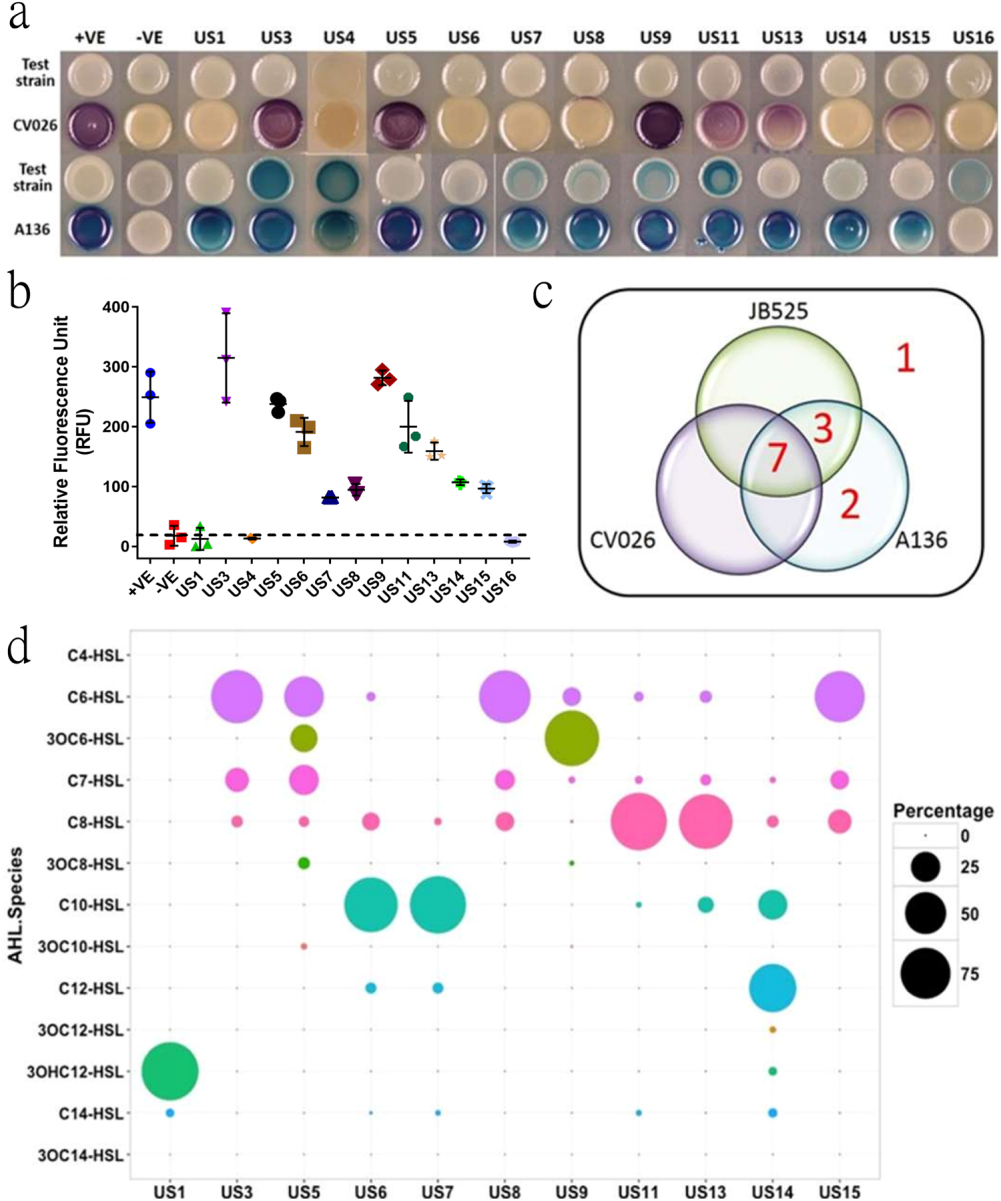
Heterologous expression of metagenomic *luxI* homolog genes recovered from Ulu Pandan sludge microbial community in *Escherichia coli* Top10. Detection of AHLs synthesized by different metagenomic *luxI* clones using AHL sensitive biosensors *Chromobacterium violaceum* CV026 and *Agrobacterium tumefaciens* A136 (**a**) or *E. coli* JBA525 (**b**). Distribution of metagenomic *luxI* clones according to their capacity to activate different AHL-sensitive biosensors (**c**). Chemical characterization and quantification of AHL species using HPLC–MSMS (**d**). The relative quantity of different AHL species synthesized by the individual clone is indicated by the bubble size. US: Ulu Pandan Synthase (Metagenomic *luxI* clone); +VE: positive control; −VE: negative control. Data are presented as mean ± SD.

The ability of the putative *luxI* homologs to synthesize AHLs was evaluated using three AHL biosensors, *A. tumefaciens* A136, *C. violaceum* CV026, and *E. coli* JB525. All *luxI* homologs, except for US16, activated at least one of the three biosensors (Fig. 1A–C). Seven homologs (US3, US5, US8, US9, US11, US13, and US15) activated all three biosensors, while three genes (US6, US7, and US14) activated both the A136 and JB525 biosensors. Homologs US1 and US4 activated only the A136 biosensor. To further characterize the AHLs produced by the *luxI* homologs, we extracted the AHLs from overnight culture and subjected them to LC–MSMS for AHL identification and quantification (Fig. 1D). US1 and US14 predominantly produced long-chain AHLs such as 3OHC12-HSL and C12-HSL, respectively, while US6 and US7 mainly produced C10-HSL. Homologs US3, US5, US8, US9, and US15 produced C6-HSL (3OC6-HSL for US9) as the major AHL species, however, other intermediate-chain AHLs including 3OC6-HSL, C7-HSL, and C8-HSL were also detected at lower amounts. Homologs US11 and US13 also produced C8-HSL. The LC–MSMS results for US4 and US16 did not match any of the 13 AHL standards used. No C4-HSL were observed for any of the putative *luxI* homologs. A potential limitation of the heterologous expression of LuxI is that the AHL produced might be different from the original host due to the different fatty acid pools^28,29^.

The putative functionality of *luxR* homologs was assessed based on the conserved residues in the acyl-binding domain (W66, D79, P80, W94, and G121) as well as the DNA binding domain (E187, L191, and G197)^30^. The five conserved residues in the acyl-binding domain were present in all nine of the paired *luxR* genes as well as four of the *luxR* solo genes. The remaining 17 *luxR* solo genes carried one to three missense mutations in the five conserved residues. The three key residues in the DNA-binding domain were fully conserved in 24/30 of the *luxR* homologs, while the remaining six homologs carried a missense mutation in residue E187 or L191 (Supplementary Table 5). Most of the conserved residues are present in the putative *luxR* homologs, which suggests that they are likely to be functional.

### Phylogenetic tree of the putative *luxIR* genes and their taxonomic relationships

**Fig. 2:**
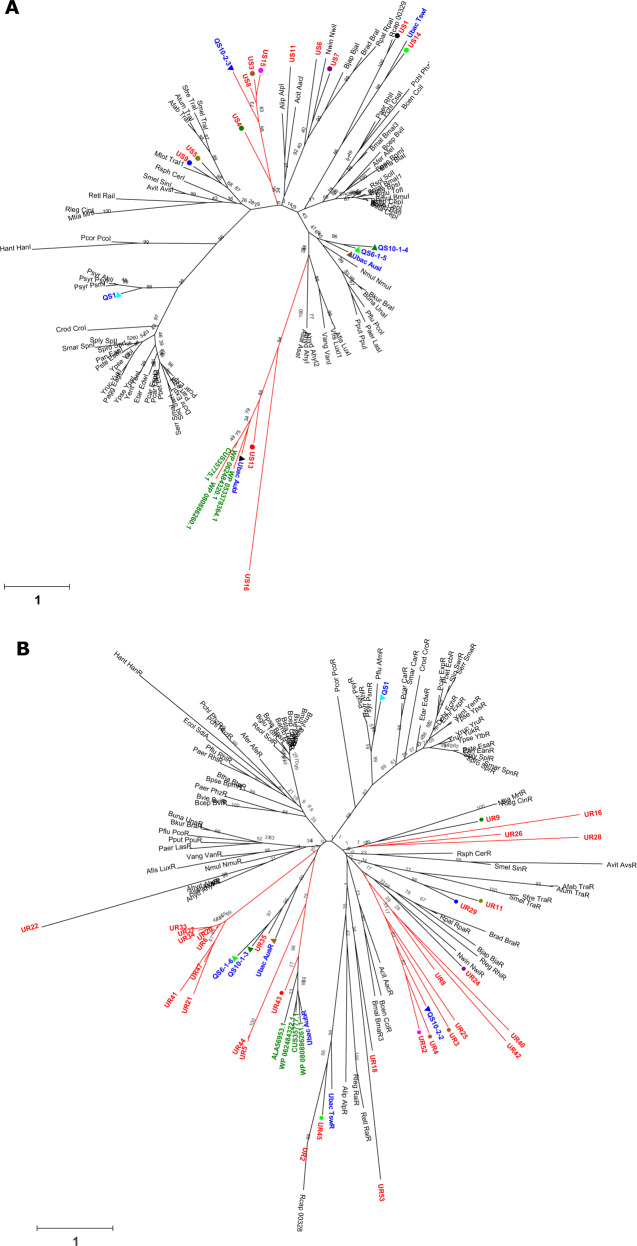
Phylogenetic tree illustrating the evolutionary relationship of the metagenomic *luxI* and *luxR* constructed using the maximum-likelihood method. Phylogenetic analysis was performed by comparing the amino acid sequences of the metagenomic *luxI* genes (**a**) and metagenomic *luxR* genes (**b**) to 80 and 85 functionally verified *luxI* and *luxR* that were previously reported in the literature, as well as four *luxI*/*R* pairs obtained from *Nitrospira*. The *luxI*/*R* homologs verified in pure culture studies, metagenome studies, and other *Nitrospira* were written in black, blue, and green, respectively, while the putative *luxI*/*R* homologs identified in this study was written in red. The paired *luxI*/*R* homologs were indicated by symbols with the same shape and color.

To investigate the phylogenetic relationship between the putative *luxIR* from sludge community and the characterized *luxIR*, we compared the amino acid sequence of the putative *luxIR* genes to 80 and 85 verified *luxI* and *luxR* homologs, respectively, which derived from pure cultures and metagenomic studies, as well as four *luxI*/*R* pairs obtained from *Nitrospira* (Supplementary Table 2). The phylogenetic trees were constructed using the neighbor-joint and maximum-likelihood method. As both methods yielded similar results, only the neighbor-joint tree is shown here (Fig. 2).

Of the 13 putative *luxI* genes, six homologs formed two distinct clades in the *luxI* tree. This includes the *luxI* homologs US3, US4, US8, and US15 which formed a unique branch along with a *luxI* gene from a previous metagenome study^19^. Another distinct clade was formed by US13 and US16 along with *aubI*, a *luxI* homolog found in *Nitrospira*, as well as other *luxI* from *Nitrospira* lineage II^21,31^. The remaining seven *luxI* homologs were distributed across different clades of verified *luxI* homologs. This includes the paired *luxI* homologs US1 and US14 which are related to a rare *luxI* homolog from *Rhodobacter capsulatus* that produced unusually long AHL, US5, and US9 which is associated with TraI-class AHL synthase, as well as US7 and an unpaired *luxI* US6 which are related to *nwiI* from *Nitrobacter*. The putative *luxR* genes were distributed across four clades in the *luxR* phylogenetic tree, which consists of one clade formed solely by the putative *luxR* solos. The remaining homologs were distributed in different branches of the *luxR* tree. Most of the paired *luxR* homologs clustered similarly to their *luxI* counterpart, suggesting a co-evolutionary relationship between the two proteins. For example, UR2 and UR45 (paired with US1 and US14, respectively) were clustered with the *luxR* homolog of *R. capsulatus*, UR11 and UR29 (paired with US5 and US9, respectively) associated with TraR class *luxR* homologs as well as UR24 (paired with US7) related to the *nwiR*. Notably, UR43 (paired with US13) was also found to be closely associated with the *aubR* as well as other *luxR* homologs from *Nitrospira*, suggesting that the US13/UR43 *luxIR* pair originated from a *Nitrospira* sp.

### Taxonomic origin of AHL producers and AHL responders

**Fig. 3:**
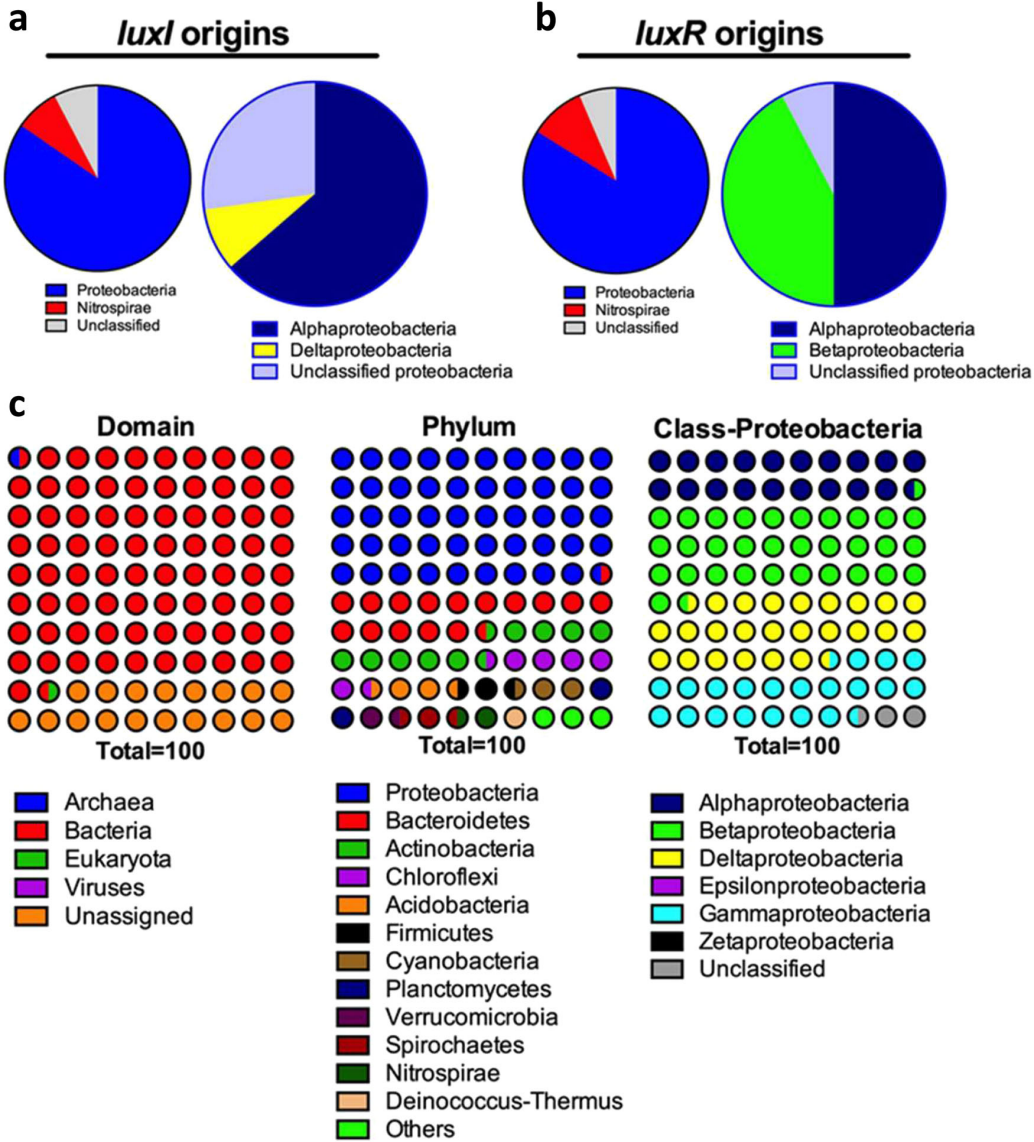
Taxonomy association of AHL signal and AHL response genes in the activated sludge community. Pie charts show the taxonomic distribution of AHL signaler (**a**) and AHL responder (**b**) at the phylum level (left) and within the Proteobacterial class (right) based on the contig ORFs analysis. The taxonomic distribution of the total community at domain, phylum, and Proteobacterial classes (**c**) based on the ORFs analysis of metagenomic contigs.

To identify the *luxI* (AHL producer) and *luxR-*harboring (AHL responder) bacteria in the sludge community, we analyzed the contigs containing the putative *luxIR* genes for their taxonomic affiliation. At the phylum level, both AHL producing and AHL responding bacteria were represented by *Proteobacteria* (84.6% and 83.9%, respectively), *Nitrospirae* (7.7% and 9.7%, respectively), and unclassified bacteria (7.7% and 6.4%, respectively) (Fig. 3A and B). The proteobacteria-related AHL producers were 63.3% *α-proteobacteria*, 9.1% *δ-proteobacteria,* and 27.3% unclassified proteobacteria. In contrast, the proteobacteria-related *luxR* homologs were assigned as 50% *α-proteobacteria*, 42.3% *β-proteobacteria,* and 7.7% unclassified proteobacteria. Although QS has been commonly reported in the *γ-proteobacteria*, no *luxI* or *luxR* genes mapped to this group of bacteria. The ORFs from all the metagenome contigs were analyzed to determine the taxonomic distribution of the whole community. This revealed a roughly equal distribution of *α-proteobacteria* (19.71%), *β-proteobacteria* (31.64%), *γ-proteobacteria* (20.91%), and *δ-proteobacteria* (25.14%) in the proteobacterial community (Fig. 3C), suggesting that the *γ-proteobacteria* are present in the sludge community, despite the lack of QS genes found here associated with this clade.

### AHL QS regulon in *Nitrospira*

The results above clearly suggest that a *Nitrospira* species in the activated sludge encodes the machinery to produce and respond to AHLs (US13 and UR43). Indeed, AHL QS genes of *Nitrospira* were first reported in 2012^21^, however, since the organism has been difficult to purify and cultivate for laboratory studies, the QS regulon of this organism remains to be explored. Given that *Nitrospira* plays a major role in the nitrification process in the activated sludge^32^, the AHL-controlled genes of this organism were investigated here using a machine-learning approach to screen for the presence of *lux* box in *Nitrospira* contigs to identify potential AHL QS-regulated genes. Using this approach, 219 genes were predicted to be AHL regulated (Supplementary Table 4).

**Fig. 4:**
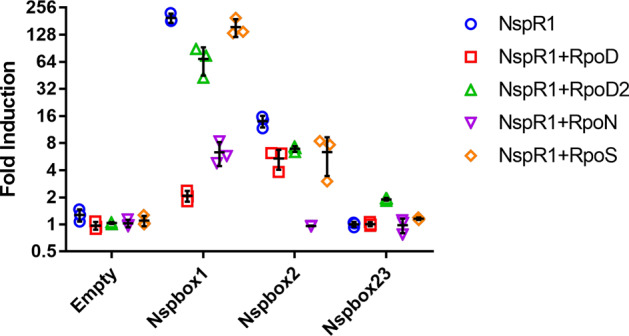
Regulation of NspR1 on the predicted lux-box containing promoters with and without the co-expression of Nitrospira sigma factors. The screening was performed in *E. coli* TOP10 transformed with pPROBE-gfp(ASV) containing the predicted lux-box containing promoters (Nspbox) and pUCP22-NspR1 or pUCP22-NspR1 carrying the Nitrospira sigma factor gene. Relative fold induction was calculated by measuring the fold induction of GFP signals of the induced sample (added with 1 mM C8-HSL) to uninduced control (added with DMSO). Data are presented as mean ± SD.

Of those, 61 genes were selected based on the relative location of the *lux* box (intergenic vs. intragenic) and the associated *p*-value for the verification of NspR1 regulation. To determine if the genes are indeed regulated by the *Nitrospira luxR* homolog, which we designated as NspR1, these 400 bp *lux* boxes containing upstream regions (designated as Nspboxes) were cloned upstream of a promoterless green fluorescence protein gene (*gfp*) in an *E. coli* host that also expresses NspR1. Since the associated *luxI* homolog, now designated as *nspI*, was shown to primarily produce C8-HSL, the reporter strain was incubated with this AHL, and GFP expression was monitored. A cut-off of >1.5 fold increase in GFP fluorescence intensity after C8-HSL induction was used to indicate positive induction. Of the 61 Nspboxes tested, Nspbox1 and Nspbox2 were associated with a 196.6 fold (*p* < 0.0001) and 14.0 fold (*p* < 0.0001) increase in GFP production, respectively, relative to the no Nspbox control after AHL addition (Fig. 4). No GFP induction was observed for the remaining Nspbox clones (Supplementary Table 6). Notably, Nspbox1 and Nspbox2 are overlapping in the upstream regions of two divergently transcribed genes that shared a common *lux* box sequence (ACCTGGCGGTTCCGCCAGGT). In Nspbox1, the *lux* box is located 125 bp upstream to a gene encoding a hypothetical GGDEF/EAL domain-containing protein, while the *lux* box is located 81 bp upstream to the cognate AHL synthase gene *nspI* in Nspbox2.

While two of the Nspbox regions responded to C8-HSL and required NspR1 for activity, the remaining 59 putative promoters showed no activity. It has previously been shown that promoter activity can be enhanced by co-expressing a host sigma factor^22^. Therefore, we identified and co-expressed four *Nitrospira* sigma factors, including two *rpoD* (designated as *rpoD* and *rpoD2*, respectively), one *rpoN,* and one *rpoS* genes in the *E. coli* screening host. With this, an additional target, Nspbox23 was upregulated (1.9 fold, *p* < 0.05) when *rpoD2* was co-expressed (Fig. 4). Interestingly, co-expression of these sigma factors did not enhance expression from the Nspbox1 or Nspbox2 clones or, as in the case of RpoN, significantly reduced activity (Fig. 4).

### DNA binding of NspR1

**Fig. 5:**
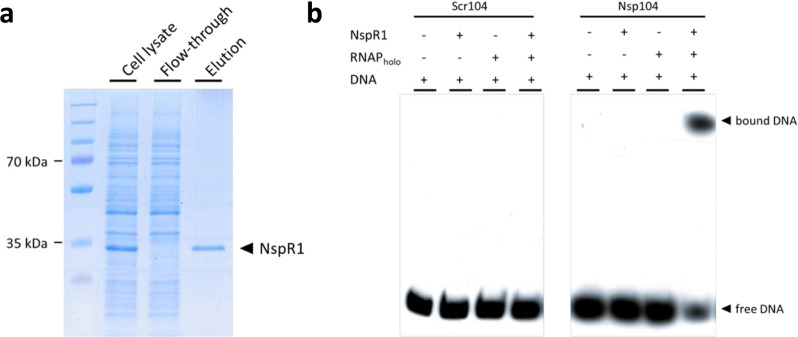
Purification and DNA-binding of NspR1. Purification of soluble NspR1 using Ni-NTA column (**a**). Soluble 6xHis-tagged NspR1 was obtained by overexpressing the protein in the presence of 5 μM C8-HSL at 17 °C for 16 h. DNA-binding of purified NspR1 to the DNA probe containing Nspbox1 sequence, Nsp104 and scramble control Scr104, in the presence and absence of *E. coli* RNA polymerase holoenzyme (**b**). The EMSA gel for both Scr104 and Nsp104 samples were derived from the same experiment and then they were processed in parallel.

To further support the role of NspR1 as an AHL-dependent DNA-binding protein, the hexahistidine-tagged NspR1 was overexpressed and purified. Soluble NspR1 was successfully obtained by incubation at 17 °C and the addition of 5 μM C8-HSL during the protein induction phase (Fig. 5A). Two biotinylated DNA probes, Nsp104 and Scr104 were designed. Nsp104 encodes the common *lux* box sequence (ACCTGGCGGTTCCGCCAGGT) of Nspbox1 and Nspbox2, as well as the 40 and 44 bp upstream and downstream of the *lux* box, respectively. Scr104 shared the same sequence as Nsp104 except with the *lux* box replaced with a randomized nucleotide sequence (GATACGGCTTGGTGATCGCG).

EMSA was performed by incubating NspR1 with the Nsp104 or Scr104 in the presence of C8-HSL. However, no DNA binding to Nsp104 or Scr104 was observed (Fig. 5B). On the other hand, replacing the purified NspR1 with the cell lysate of NspR1-overexpressing BL21 (Supplementary Fig. 1) or adding a BL21 cell lysate (background strain) to the DNA-binding reaction containing purified NspR1 resulted in an NspR1-specific band shift of Nsp104 but not Scr104 (Supplementary Fig. 2). This result suggests that a co-binding partner(s) may be required for NspR1-mediated DNA binding in the presence of AHL. To identify the co-binding partner required, we co-incubated purified NspR1 and RNAP holoenzyme from *E. coli* with the DNA probes induced an NspR1-specific band shift of Nsp104, which was not observed when the DNA was incubated with NspR1 or RNAP alone (Fig. 5B). Also, replacing the RNAP holoenzyme with a core enzyme (without sigma factor) does not prevent the co-binding with NspR1 to Nsp104 (Supplementary Fig. 3), indicating that DNA-binding involved the interaction between NspR1 and the core unit of RNAP but not with the sigma factor.

## Discussion

The role of QS signaling in complex communities has been challenging to study, in part due to the difficulties in identifying individual QS producers and responders. This has typically been approached by either culturing individual species and testing them as pure cultures or through shotgun cloning^19–21^. However, these methods were limited by the fact that many organisms are difficult to culture, and shotgun cloning may result in genes not being expressed due to their position in vector or a lack of required regulatory proteins (e.g. native sigma factors, RNA polymerase, etc.). As a result, these approaches often identify low numbers of QS genes. In contrast, metagenomic sequencing studies have revealed a larger number of QS genes, although they may not capture and verify that the genes are functional^24,25,33^. In this study, we identified 13 putative *luxI* and 30 putative *luxR* homolog genes (representing 0.0012% and 0.0028% of total ORFs) in a complex sludge community from the UP WWTP. For comparison, it was found that AHL QS genes represent 0.0015% and 0.046% in the metagenome extracted from activated sludge and biofilm from membrane bioreactor, respectively^34^, while another study reported no QS genes were found in the microbial community from terephthalate-degrading wastewater bioreactor^25^. In addition, we functionally verified the putative *luxI* genes using AHL biosensor strains and LC–MS/MS, while for *luxR*, using conserved domain analysis. Except for US16, all of the *luxI* genes activated at least one of the AHL biosensors, demonstrating they are functional and most *luxI* produced intermediate chain (C6 and C8) AHL. In our previous study, the concentration of intermediate-chain AHLs, e.g. 3OC6-HSL, C6-HSL, C8-HSL, and 3OC8-HSL correlated with the transition of floccular sludge into granular sludge as well as EPS production^13^. Interestingly, many of the *luxI* homologs identified in this study were able to produce three out of the four key AHLs, suggesting their potential role in stimulating the granulation process of the sludge community.

One *luxI* and three *luxR* homologs found in this study were linked to *Nitrospira*, which is recognized as the key NOB in both natural and engineered habitats^35,36^. AHL QS genes in *Nitrospira* were first described in 2012^21^ and to date, relatively few studies have been reported for *Nitrospira* AHL QS^26,31,34,37^. The AHL QS in *Nitrospira* was reported in *N. defluvii* from *Nitrospira* lineage I as well as *N. moscoviensis*, *N. japonica*, *N. inopinata*, and *N. nitrifican* from lineage II which includes the comammox group that is capable of complete ammonia oxidation^21,31^. Because we were unable to recover full-length 16S rRNA genes or achieve sufficient genome coverage, we are unable to resolve the lineage of the *Nitrospira* harboring the AHL QS genes identified in this study. Based on the BLAST search, the majority of the ORFs found on the *Nitrospira* contigs were mostly related to *N. defluvii* from lineage I. In addition, we also did not detect the *amoA* gene in the contigs assigned to the *Nitrospira* contigs. This would suggest that the species identified here is not a Comammox organism. A hybrid Illumina and Nanopore long-read sequencing approach could be performed in the future to improve the genome recovery in order to resolve the lineage of the *Nitrospira* identified in this study.

Based on sequence comparison, all of the *Nitrospira* QS genes were closely related regardless of lineage suggesting that it is likely that they were inherited from ancient common ancestors with *Proteobacteria* rather than acquired through horizontal gene transfer event^21^. Whether QS regulates nitrification and the survival fitness in this bacterium remained unclear. Given its important role in the nitrogen removal process in WWTP and scarce knowledge on the QS system in *Nitrospira*, we decided to further study the cognate pair of *Nitrospira* AHL QS gene US13 and UR43, which were later designated as *nspI* and *nspR1*, respectively. NspI was most closely linked to *N. defluvii* and produced C8-HSL as the dominant AHL species. The AHL species reported in other *Nitrospira* include C8-HSL (*N. moscoviensis*) and C12-HSL (*N. japonica* and a metagenomic clone of *N. defluvii*)^21,31,37^. NspR1, on the other hand, was found to regulate the expression of its cognate AHL synthase NspI and a GGDEF/EAL domain-containing hypothetical protein in a GFP-reporter assay when C8-HSL was added. The upregulation of *nspI* gene by its own cognate LuxR suggests that the AHL QS in this *Nitrospira* spp. is also subjected to the autoinduction feedback system, which is commonly observed in many other QS-proficient bacteria^2,38^. GGDEF/EAL-domain-containing proteins are known to regulate a diverse set of cellular functions including biofilm formation and bacterial virulence^39^ via modulating the concentration of intracellular cyclic-di-GMP in response to environmental cues such as oxygen and nitric oxide^40,41^. Hence, it is possible that the AHL QS in *Nitrospira* spp. may indirectly regulate multiple downstream genes via the cyclic-di-GMP pathway or fine-tuning the gene expression by integrating of QS signal and local environmental cues. The two genes are oppositely oriented and shared a common *lux* box sequence (ACCTGGCGGTTCCGCCAGGT) in their promoter sequence, indicating that the transcriptional regulatory effect of NspR1 is bi-directional. The results of the GFP-reporter assay are further supported by the EMSA result which demonstrates the binding of NspR1 to the Nsp104 probe which carries the common *lux*-box sequence, confirming that this *lux* box is truly regulated by the NspR1. All of the other putative lux boxes had at least two nucleotide differences and thus may suggest that the *lux* box described above is the true recognition sequence for NspR1. Interestingly, the NspR1 only bound the *lux* box in the presence of RNA polymerase (Fig. 5). Although RNA polymerase has been reported to interact with Proteobacterial LuxR homologs, such as LasR, LuxR, and TraR to facilitate the expression of target genes^42,43^, the DNA binding does not require the presence of RNA polymerase^44–47^. Hence, the requirement of RNA polymerase for DNA binding might be unique to the *Nitrospira* LuxR.

Co-expression of the native host’s sigma factor has been shown to facilitate the recognition of the foreign promoter in the screening host^22^. With the co-expression of one of the *rpoD* genes (*rpoD2*), we identified an additional Nspbox (Nspbox23) under the regulation of AHL QS. Nspbox23 contains a palindromic *lux* box-like sequence (CACTGGACGAGTGTACAGTT) and is located 187 bp upstream to a gene encoding putative helix-turn-helix domain-containing protein, suggesting its potential role as a transcription regulator. Sigma factors RpoS and RpoN were reported to co-regulate the QS regulon in *Pseudomonas aeruginosa* and *Burkholderia pseudomallei*^48–51^. However, in this study, we did not identify any additional Nspbox induced with the co-expression of the *Nitrospira rpoS* and *rpoN* gene. Instead, the co-expression of *Nitrospira* sigma factors affect the AHL QS induction of Nspbox1 and Nspbox2 at varying degree. In particular, Nspbox2 which regulates the expression of the AHL synthase *nspI* was completely suppressed by co-expression of the *Nitrospira rpoN* gene. RpoN has been shown to regulate gene expression under nitrogen-limiting conditions and also to regulate diverse functions such as virulence and motility^52–54^. Given that nitrogen serves as the major energy source for NOB, it is possible that QS in *Nitrospira* is repressed under nitrogen-limiting conditions. These findings suggest that C8-HSL QS may play a role in regulating the nitrification process to acquire energy more efficiently and to compete for the nitrogen resources in a mixed community. Indeed, it was reported previously that C8-HSL could accelerate ammonium oxidation rate by the anaerobic ammonium oxidation (ANAMMOX) communities dominated by *Candidatus Brocadia fulgida*^55^ or *Candidatus Jettenia caeni*^56^ and modulate nitrogen oxide fluxes in *N. winogradskyi*^57^.”

This study shows that a combination of bioinformatics and in vitro functionality tests can be applied to study AHL-mediated QS in a complex community such as an activated sludge system. Our results show that there are many novel *luxI* and *luxR* homolog genes found in the activated sludge and we expect that the same will also be observed in other complex ecosystems. We demonstrated the functionality of both the *luxI* and *luxR* gene from a QS-proficient *Nitrospira* that is present in the sludge metagenome and describe an unusual DNA-binding behavior that was not observed for other LuxR receptors. This provides valuable insight into the molecular mechanism of AHL QS in *Nitrospira*. Further work would include investigating the downstream regulatory outcomes of QS in this organism, such as the implications for AHLs to regulate the expression of c-di-GMP modifying enzymes and the associated genes and phenotypes.

## Materials and methods

### Generation of the activated sludge metagenome

Activated sludge was collected from UP wastewater reclamation plant, Singapore, and stored at −80 °C prior to DNA extraction. Total DNA was extracted using the FastDNA™ SPIN Kit for Soil (MP Biomedical) and sequenced. Firstly, 10 runs of Roche 454 data and 1 lane of 130 bp Illumina SE reads were used for assembly using the Roche GS De Novo Assembler (Newbler v2.5.3). This initial assembly generated 1,092,203 contigs with an N50 contig size of 1168 bp. From these contigs, we simulated 4.5 million 2 × 100 read pairs with an insert size of 1 kbp, and 0.8 million 2 × 100 read pairs with an insert size of 5 kbp. A second sequencing run was performed to obtain paired-end reads from Illumina short insert size libraries (300 bp) and was assembled into contigs using SOAPdenovo (v1.0.5)^58^. All available paired-end and simulated 454 mate-paired reads were mapped to these contigs to generate scaffolds and gaps closed using Gapcloser (v1.12)^58^. The resulting metagenome assembly comprising 3.7 Gb was deposited to GenBank under BioProject ID 226634.

### Identification of putative *luxIR* genes in the sludge metagenome

MetaGeneMark (v2.8) was used for identifying protein-coding regions. A total of 1,056,488 open-reading frames (ORF) were found in the 220,511 contigs (≥1 kb) of the sludge metagenome. The KO groups were used as the reference database for the AHL receptor (K07782, K18098, or K18099) and the AHL synthase (K13060, K13061, K13062, or K18096). Using BLASTX, any ORF mapped to QS-related KOs with a score higher than for other KOs were classified as a potential QS gene. The putative *luxI* and *luxR* genes identified using this KO approach were independently validated using Pfam for the presence of the autoinducer synthase conserved domain (Pfam00765) for LuxI, and the autoinducer-binding domain (Pfam03472) as well as the GerE domain (Pfam00196) for LuxR.

### Bacterial culture and strain construction

*E. coli* TOP10 and *E. coli* BL21 were cultured in Luria-Bertani (LB) broth at 37 °C, 200 rpm, unless specified otherwise. Kanamycin and gentamycin were supplemented at a final concentration of 50 μg/ml whenever appropriate. Plasmids and primers used to construct screening strains and expression strains are given in Supplementary Table 1. For AHL characterization, the putative *luxI* genes were cloned from the sludge metagenomic DNA into the expression vector pUCP22-Not1 and expressed in *E. coli* TOP10. For the GFP-reporter assay, the 61 predicted *Nitrospira lux* box-containing upstream regions (designated as Nspboxes) were amplified from the sludge metagenomic DNA and cloned into the screening vector pPROBE-gfp(ASV), upstream of the promoter-less *gfp* gene. The *Nitrospira luxR* homolog UR43 (later designated as NspR1) was amplified from the sludge metagenomic DNA and cloned into pUCP22-Not1 to monitor GFP as a proxy for expression. A two-step cloning method was used to clone the four *Nitrospira* sigma factors, *rpoD*, *rpoD2*, *rpoN,* and *rpoS*. The sigma factor genes from the sludge metagenomic DNA were first cloned into pUCP22-Not1 downstream to the *Plac* promoter. The second cloning step involved amplifying the sigma factor gene, along with the *Plac* promoter into the NspR1-expressing plasmid pUCP22-NspR1.

### AHL biosensor assay

AHL production was monitored using *Agrobacterium tumefaciens* A136 by spotting 10 μl of an overnight culture of *A. tumefaciens* A136 and *E. coli* TOP10 expressing the putative *luxI* homolog genes 5 mm apart on an ABT indicator agar^59^ containing 50 μg/ml of 5-bromo-4-chloro-3-indolyl-b-d-galactopyranoside (X-gal). The plate was incubated at 30 °C for 48 h for color development. Similarly, AHL production was characterized using *Chromobacterium violaceum* CV026, except that LB agar was used instead of ABT. *E. coli* JB525 was also used to determine AHL production by mixing an equal volume of five times diluted *E. coli* JBA357 overnight culture with *E. coli* TOP10 expressing the putative *luxI* homolog gene and incubated at room temperature with constant shaking at 200 rpm for 4 h before examination by confocal laser scanning microscope (Zeiss LSM 710, Carl Zeiss Pte. Ltd., Singapore) at excitation and emission wavelengths of 488/522 and 535 nm, respectively.

### Identification and quantification of AHLs produced by the putative *luxI* gene

AHL identification and quantification were performed according to Tan et al.^13^. Briefly, AHLs from the supernatant of an overnight culture was extracted with dichloromethane and resolved by high-performance liquid chromatography–tandem mass spectrometer system (Shimadzu LCMS8030, Shimadzu) using a Shim-pack XR-ODS C18 column under a linear gradient (40–95%) of solvent B (methanol with 0.1% formic acid) and solvent A (25 mM ammonium formate with 0.1% formic acid) at a flow rate of 0.3 ml/min. The specific retention time, precursor ion *m*/*z*, and two transition ions of each sample were monitored and matched to the profile of 13 AHL standards ranging from 0.5 to 200 μg/l^13^. Blank injections were performed at the intervals of sample injections to avoid sample carryover.

### Phylogenetic analysis of putative *luxIR*

The amino acid sequences of the putative *luxI* and *luxR* homologs were compared to 80 and 85 biochemically verified *luxI* and *luxR* homologs, respectively, from axenic cultures and six metagenomic studies, as well as four *luxI*/*R* pairs coming from *Nitrospira* (Supplementary Table 2) for phylogenetic inference. Multiple sequence alignment was performed using MUSCLE and phylogenetic analyses were conducted with MEGA X^60,61^. The evolutionary relationships of *luxI* and *luxR* homolog genes were inferred using the neighbor-joining method using the JTT model, as well as the maximum-likelihood method using the LG model^62,63^. The resulting phylogenetic tree was constructed using MEGA X^61^.

### Taxonomic annotation of putative *luxIR* homologs

Contigs containing putative *luxIR* homologs were compared against the NR database using BLASTP^64^ (with *E* < 1e−02) and were taxonomically and functionally annotated using a combination of a lowest common ancestor (LCA) and consensus approaches^65^. Briefly, for each protein, we set Sbest as the bitscore of the best BLAST match and selected all with a bitscore higher than 0.95 Sbest. Using the functional annotation of these selected BLAST matches, we applied a majority-rule consensus approach to determine the query protein’s function. We then computed the LCA of all species in the selected BLAST matches to determine their potential taxonomic origin. Similar outcomes were obtained based on the JGI-IMG annotation. To allow for better comparison, the taxonomic composition of the total sludge community was determined using the same BLASTP and LCA approach.

### Identifying *Nitrospira* contigs

Contig identity was determined using an alignment-based approach. The metagenome contigs (220, 511 scaffolds with ≥1000 bp) were split into 1 kbp fragments and BLASTX analysis was performed using DIAMOND^66^ against the NR database. The contigs were taxonomically classified based on the default MEGAN analysis setting^67^ using a combination of a LCA and majority-rule consensus approaches^65^. We further refined the MEGAN outputs (i.e., putative Nitrospira contigs) with empirical parameters outlined below to minimize false positive and false negative taxonomic assignments of the contigs, especially for contigs with ORFs <6. The majority-rule consensus was applied in all cases^65^. A contig was annotated as *Nitrospira* if one of the following criteria were met: (i) >50% of the total ORFs are classified as *Nitrospira* and if the total number of ORFs are >6; (ii) >60% of the total number of ORFs are classified as *Nitrospira* and if the total number of ORFs are between 4 and 6; or (iii) 100% of the total ORFs are classified as *Nitrospira* and if the total number of ORFs are <3.

### Prediction of AHL-regulated genes in *Nitrospira*

After construction and annotation of the *Nitrospira* genome bin, the upstream region (400 bp) of each ORF was extracted using regulatory sequence analysis tools (RSAT)^68^. Potential *lux* box sequences in the upstream regions were identified using MEME-FIMO^69^, which was trained with a library consisting of 54 highly conserved, previously reported *lux*-box sequences (Supplementary Table 3). Three criteria were applied in the search which were: (i) 20 bp nucleotide length, (ii) imperfect palindromic sequence, and (iii) a default *p-*value of <0.0001 was chosen. The upstream regions were further analyzed for the (i) relative location of *lux-*box (intergenic vs. intragenic), (ii) the presence of −10 and −35 promoter sequence, (iii) a Shine–Dalgarno sequence, and (iv) function of the associated gene.

### GFP-reporter bioassay

The selected *lux-*box containing upstream regions, designated as Nspbox (Supplementary Table 4), were cloned into screening vector pPROBE-gfp(ASV) and transformed into *E. coli* TOP10 expressing the *Nitrospira luxR* homolog UR43 (designated as NspR1) only, or co-expressing NspR1 with one of the *Nitrospira* sigma factors (*rpoD*, *rpoD2*, *rpoS*, and *rpoN*) from the expression vector pUCP22-Not1. To check if NspR1 activates the Nspboxes in the presence of AHL, overnight cultures were diluted 10 times with complete M9 medium and aliquoted into 96-well plates, 200 μl per well. The cognate *N*-octanoyl-l-homoserine lactone (C8-HSL) was then added at a final concentration of one micromolar. For controls, an equal volume of DMSO instead of C8-HSL was added to the cultures. At least three biological replicates were performed for AHL-induced samples and DMSO control each. The cultures were incubated at 37 °C for 24 h before GFP fluorescence measurement using NanoQuant Infinite M200 microplate reader (TECAN) and normalized to OD_600_ and uninduced control. Graphs and statistical analysis were performed using GraphPad Prism 6. A two-way anova followed by Dunnet’s multiple comparison test was used to compare the upregulation of the Nspboxes to the empty plasmid (Empty) control.

### NspR1 purification

NspR1 purification was performed according to Corral’s protocol^70^ with the following modification. An overnight culture of *E. coli* BL21 pNIC28-BSA4.6xHis-NspR1 was diluted 1000× into 100 ml of fresh LB medium containing 50 μg/ml kanamycin and 5 μM C8-HSL to solubilize the LuxR^71,72^. NspR1 expression was induced by the addition of 0.1 mM isopropyl β-d-1-thiogalactopyranoside (IPTG) at OD_600_ 0.5 and culture was incubated at 17 °C for 16 h. To harvest protein, the induced culture was centrifuged at 8000 × *g* for 5 min and the pellet was resuspended in purification binding buffer (20 mM Tris–HCl pH 7.4, 500 mM NaCl, and 10 mM imidazole). The cells were lysed by sonication at 40% amplitude for 10 cycles of 10 s with 30 s interval between each cycle. Insoluble fractions were removed by centrifugation at 21,000 × *g* for 30 min.

HisPur™ Ni-NTA Superflow Agarose (Thermo Fisher Scientific, USA) was used to purify histidine-tagged NspR1. Briefly, the soluble fraction of cell lysate was incubated with a one-milliliter bed volume of resin (pre-equilibrated with purification binding buffer) in a 5 ml centrifuge column for 1 h with constant mixing. The column was centrifuged at 700 × *g* for 2 min and the flow-through was discarded. The resin was washed three times with wash buffer (20 mM Tris–HCl pH 7.4, 500 mM NaCl, 50 mM Imidazole). A total of 4 ml elution buffer (20 mM Tris–HCl pH 7.4, 500 mM NaCl, 300 mM Imidazole) was added to the resin and incubated for 10 min prior to centrifugation at 700 × *g* for 2 min. Finally, Vivaspin500 (Sartorius, Germany) was used to remove the imidazole and concentrate the protein sample into a storage buffer (20 mM HEPES pH 7.5, 500 mM NaCl, 10% glycerol, 2 mM TCEP) supplemented with 5 μM C8-HSL and 0.3% Protease Inhibitor Cocktail (Sigma-Aldrich, USA). The protein concentration was quantified using Qubit™ Protein Assay Kit.

### DNA binding activity of NspR1

The EMSA protocol was adapted from Schuster et al.^72^. The DNA binding reaction was performed in a binding buffer (10 mM Tris–HCl pH 7.5, 50 mM KCl, 10 mM DTT, 10 ng/μl poly-dIdC, 100 ng/μl BSA, 5% glycerol, 1 mM EDTA, and 5 μM C8-HSL) containing 10 fmol of biotinylated DNA probes. The DNA-binding proteins, which include 300 ng of purified NspR1, 1 μg of *E. coli* BL21 cell lysate, 1 U of *E. coli* RNA polymerase holoenzyme (NEB, USA), or 1 U of *E. coli* RNA polymerase core enzyme (NEB, USA) were added where indicated. As a negative control, water was added to the binding reaction instead of the DNA-binding protein. The mixtures were incubated at room temperature for 60 min. After incubation, 5 μl of Novex Hi-Density TBE loading dye (Thermo Fisher Scientific, USA) was added to the sample and loaded into a DNA retardation gel (Thermo Fisher Scientific, USA). To preserve the binding complex, the gel was electrophoresed at 100 V for 100 min at 4 °C in 0.5× TBE. Once completed, the DNA was transferred onto a pre-equilibrated nylon membrane using a wet transfer system at 100 V for 60 min in 0.5× TBE buffer at 4 °C. The DNA was cross-linked onto the membrane by exposing the membrane with ultraviolet light (254 nm) at 1200 kJ for 30 s. The biotinylated DNA probes were detected using the LightShift Chemiluminescent EMSA Kit (Thermo Fisher Scientific, USA) according to the manufacturer’s instruction.

### Reporting summary

Further information on research design is available in the Nature Research Reporting Summary linked to this article.

## Supplementary information

Supplementary Information

Reporting Summary

## Data Availability

Sludge metagenome was deposited in GenBank under BioProject ID 226634. All other data used in this study, including raw data, are available in NTU repository DR-NTU (Data) under 10.21979/N9/6OLO7I.

## Code availability

No custom code was used in this study. Analyses were performed using commercially or freely available software with the version and setting specified in “Materials and methods” section.
