# Supplementary material for: Functional metagenomic analysis of quorum sensing signaling in a nitrifying community
Source: NPJ Biofilms Microbiomes. 2021 Oct 28;7:79. doi: 10.1038/s41522-021-00250-3 (PMC8553950; doi:10.1038/s41522-021-00250-3)
Supplement: Supplementary file 1 — Supplementary Information [file 41522_2021_250_MOESM1_ESM.pdf]

Supplementary Table 1. List of plasmids and primers used in this study

Plasmids	Sources
Parental plasmids	
pUCP22-Not1	West SHE, et al (1994)
pPROBE-gfp(ASV)	Miller WG, et al (2000)
pNIC28-BSA4	Savitsky P, et al. (2010)
Expression of putative *luxI*	
pUCP22-Not1	
US3	this study
US4	this study
US5	this study
US6	this study
US7	this study
US8	this study
US9	this study
US11	this study
US13	this study
US14	this study
US15	this study
US16	this study
Screening of promoters regulated by Nitrospira QS	
pUCP22-Not1	
NspR1	this study
NspR1 + rpoD	this study
NspR1 + rpoD2	this study
NspR1 + rpoN	this study
NspR1 + rpoS	this study
pPROBE-gfp(ASV)	
Nspbox1	this study
Nspbox2	this study
Nspbox3	this study
Nspbox4	this study
Nspbox5.1	this study
Nspbox6	this study
Nspbox7	this study
Nspbox8	this study
Nspbox9	this study
Nspbox10	this study
Nspbox11	this study

Nspbox12	this study
Nspbox13	this study
Nspbox14	this study
Nspbox15	this study
Nspbox17	this study
Nspbox18	this study
Nspbox20	this study
Nspbox21	this study
Nspbox22	this study
Nspbox23	this study
Nspbox24	this study
Nspbox25	this study
Nspbox26	this study
Nspbox27	this study
Nspbox28	this study
Nspbox29	this study
Nspbox30	this study
Nspbox31	this study
Nspbox32	this study
Nspbox34	this study
Nspbox35	this study
Nspbox36	this study
Nspbox37	this study
Nspbox38	this study
Nspbox40	this study
Nspbox41	this study
Nspbox42	this study
Nspbox44	this study
Nspbox45	this study
Nspbox46	this study
Nspbox47	this study
Nspbox49	this study
Nspbox50	this study
Nspbox51	this study
Nspbox52	this study
Nspbox53	this study
Nspbox54	this study
Nspbox55	this study
Nspbox56	this study
Nspbox58	this study
Nspbox59	this study
Nspbox60	this study

Nspbox61	this study
Nspbox62	this study
Nspbox65	this study
Nspbox68	this study
Nspbox69	this study
Nspbox70	this study
Nspbox71	this study
Nspbox72	this study
Purification of NspR1	
pNIC28-BSA4	
NspR1	this study

Primer	Sequence (5' -> 3')
Cloning of putative *luxI* genes	
US1_kF	CTTAATGGTACCATGGCGGGTCTTCTTCTGAA
US1_sR	CTTAATGTCGACTCAGTGCATCTTGGCTGCCA
US3_kF	CTTAATGGTACCATGATCCACATCATCACCGC
US3_sR	CTTAATGTCGACTCACGCCGCTTGGGTGAGAT
US4_kF	CTTAATGGTACCATGCACCGACTCCGGCACCG
US4_sR	CTTAATGTCGACTTAGCGGACATGATCGCGGCT
US5_kF	CTTAATGGTACCATGAGCCGAAACCAACTGAC
US5_sR	CTTAATGTCGACTCAGGAGGCGTGTCCGGCTG
US6_kF	CTTAATGGTACCATGCTGAAGGTTGTAACAGG
US6_sR	CTTAATGTCGACTCAACAGACCCGCACGCAAG
US7_kF	CTTAATGGTACCATGCTGATTGTTCTTTCCGGCT
US7_sR	CTTAATGTCGACCTAGGCAGCCGTCGGCCATGA
US8_kF	CTTAATGGTACCATGATTCACATCGTAACCGCCGA
US8_sR	CTTAATGTCGACTCACGCCGCTTGTGCGAGATC
US9_kF	CTTAATGGTACCATGGCAACCTTGGAGGATGT
US9_sR	CTTAATGTCGACCTAGGCGACGCGCAATACAG
US11_kF	CTTAATGGTACCATGCTGCCCAGGGAGGATCGCTT
US11_HindIIR	CTTAATAAGCTTTCAGGCCGCAACCCGGCGCG
US13_SacIF	CTTAATGAGCTCATGCACAGGATCGATTCCGTCGATG
US13_sR	CTTAATGTCGACCTAGCCAGCCGCAGGGCCGGGTA
US14_kF	CTTAATGGTACCATGGAAAACATCACCTTCGACC
US14_HindIIIR	CTTAATAAGCTTTCATTCGGCGGCGATGGCGGCCCGG
US15_kF	CTTAATGGTACCATGATCTACATAGTATCCGAGGAAA
US15_sR	CTTAATGTCGACCTAAGCTGCGCGCGCCAGAT
US16_kF	CTTAATGGTACCATGAAAACGCAGGCAGGGTC

US16_sR	CTTAATGTCGACTTAAGCACTCTCCCAACACG
Cloning of NspR1 and *Nitrospira* sigma factors	
NspR1(27)EcoR1_F	CTTGGAATTCATGCGCAAAGGGTCGACCAC
NspR1(27)XmaI_R	CAAGCCCGGGTTACTGTGCCGTCAGGAGTC
KpnI_rpoD_F	gctcggtaccATGCCGAAACAAGAATTGCT
XbaI_rpoD_R	cgactctagaCTACAGACTTTCCACGAAAC
KpnI_rpoN_F	gctcggtaccATGAAATTGCGACTCGATCT
XbaI_rpoN_R	cgactctagaCTAGAAGAAGCGCTTCCGTT
KpnI_rpoS_F	gctcggtaccATGGCCAGACGCGCGACAGCTCTCA
XbaI_rpoS_R	cgactctagaTCATAACAGATCTCCCCGCTTCATG
KpnI_rpoD2_F	gctcggtaccATGAAAGAGTCGATGACAGTTGAGG
XbaI_rpoD2_R	cgactctagaTTACTTAATGGCGGCGAACATTTCT
XmaI_pLac_F	GATCCCCGGGggaaagcgggcagtgagcgc
HindIII_pLac_F	GATCAAGCTTggaaagcgggcagtgagcgc
HindIII_rpoS_R	cgacaagcttTCATAACAGATCTCCCCGCTTCATG
HindIII_rpoD2_R	cgacAAGCTTTTACTTAATGGCGGCGAACATTTCT
Cloning of *Nitrospira lux* box	
Luxbox1_F	GATTTCTAGACTCCGGTTCCGAACACGCCG
Luxbox1_R	GATTGGTACCCATCAGGGCCCTCCGTCACG
Luxbox2_F	GATTTCTAGAGGGGCTCGAGTACCTCGTTTTGCA
Luxbox2_R	GATTGGTACCCATGACAGACCTCCTTCGGTACGG
Luxbox3_F	GATTTCTAGAGTTCGAATCCCACCCTCTCC
Luxbox3_R	GATTGGTACCCATGCTGTGTCGATCGGCCA
Luxbox4_F	GATTTCTAGAGATCTCAAAGACAGTGATGC
Luxbox4_R	GATTGGTACCCATCGGCACCTACCCCCTCC
Luxbox5.1_F	GATTTCTAGAGCGAGGAACCCACCTCTATC
Luxbox5.1_R	GATTGGTACCCACGGTATAACCTTAGCAGA
Luxbox6_F	GATTTCTAGACACTAACGGTTGGCCGATCA
Luxbox6_R	GATTGGTACCCATAGCAGCGGCCCCGGAGA
Luxbox7_F	GATTTCTAGAGACACCAACTCAACGGCTAT
Luxbox7_R	GATTGGTACCCATGGAAGGAGTCTCCCGAG
Luxbox8_F	GATTTCTAGAGTGAAAACCCTGGGTGACAG
Luxbox8_R	GATTGGTACCCATGTCAGCGAACCGCCTGA
Luxbox9_F	GATTTCTAGAGCGGAGACCTGGCGCGGCAT
Luxbox9_R	GATTGGTACCCACGATCGTGTGCTACCTCT
Luxbox10_F	GATTTCTAGATTGCCGACCAGCACGTGTTT
Luxbox10_R	GATTGGTACCCATCCCGCTCCCTTCGCATG
Luxbox11_F	GATTTCTAGACCGCGCGTGGAGGAAATGTT
Luxbox11_R	GATTGGTACCCATCGTGACACCTCCCTTTG
Luxbox12_F	GATTTCTAGAGCCTCCTTGAATGAAATCCG

Luxbox12_R	GATTGGTACCCATAAGGCCCTCCCCGAGGA
Luxbox13_F	GATTTCTAGAGCGTACGTCCGCCCCTGGGA
Luxbox13_R	GATTGGTACCCATGGAAGCACCTCTTTGCT
Luxbox14_F	GATTTCTAGATAGACAACGGCAGGCCGGAT
Luxbox14_R	GATTGGTACCCATCATCCCTCGAGGAAATT
Luxbox15_F	GATTTCTAGAGCACAGATCGAGAGGAGTCG
Luxbox15_R	GATTGGTACCCACCGTTCATTCGGGCTAGC
Luxbox16_F	GATTTCTAGAGCAGTGACCTTGGGACGGAA
Luxbox16_R	GATTGAATTCCATGAGCTTACGCCGGTGGT
Luxbox17_F	GATTTCTAGACGAGATCTTGGCGATTCAGG
Luxbox17_R	GATTGGTACCCATGGTGGCTCCTTGGTCGT
Luxbox18_F	GATTTCTAGAAGCCGTGCACCGAGATCTTT
Luxbox18_R	GATTGGTACCCATCGCTGCGCGGCACTGTA
Luxbox20_F	GATTTCTAGAGATGGAGGACTATACCGTAC
Luxbox20_R	GATTGGTACCCACGATTATCCTTTGTCTTG
Luxbox21_F	GATTTCTAGATGGGAGCAGAACGAGCTTGA
Luxbox21_R	GATTGGTACCACTGGAGACTTGGCTCCTTG
Luxbox22_F	GATTTCTAGAGAAGACGGTCCGCTCTACAT
Luxbox22_R	GATTGGTACCCATGGCGCGGTTGGGTTGTT
Luxbox23_F	GATTTCTAGACAGATCGTCGAATAGGTGCC
Luxbox23_R	GATTGGTACCCATGGTGAGGGGTCGGGACC
Luxbox24_F	GATTTCTAGACATGGCCGGCAGAACCGGCA
Luxbox24_R	GATTGGTACCCATTTAGAGGGAGCCGTCTC
Luxbox25_F	GATTTCTAGAATTGCACCACAAGGTCAGGG
Luxbox25_R	GATTGGTACCCATGATGACTCCTACTGAGA
Luxbox26_F	GATTTCTAGATACCACGAAGGCCACGAAGG
Luxbox26_R	GATTGGTACCCATAATGCACTCCTTCCCAT
Luxbox27_F	GATTTCTAGACGCCGACCTCGTGAAGGTCA
Luxbox27_R	GATTGGTACCCATTGCGCCACTCGACATCC
Luxbox28_F	GATTTCTAGAACTGTGTGAAGACCAAAGGA
Luxbox28_R	GATTGGTACCCACGACTGTCCCCCACTCGG
Luxbox29_F	GATTTCTAGATTCTCGCCTTCCTTCAGGGT
Luxbox29_R	GATTGGTACCCACTATAGAAGAGTCGATAG
Luxbox30_F	GATTTCTAGACAAGGTATTCCTGCCAGGTT
Luxbox30_R	GATTGGTACCCATGATCAAAACCTCGTTGG
Luxbox31_F	GATTGGATCCGATTCGACTATGCCAAGGGC
Luxbox31_R	GATTGGTACCCATTCGTGCCTCACGCCCAC
Luxbox32_F	GATTTCTAGACTGGGGCGTGATTCACCACT
Luxbox32_R	GATTGGTACCCATCGCCCATCCCCCTCAGG
Luxbox34_F	GATTTCTAGACGACATCGACATCACCAAGA
Luxbox34_R	GATTGGTACCCATCTCCTGGAGGCCTCCGC
Luxbox35_F	GATTTCTAGAATCTCGGCCTCACCGTGGCG

Luxbox35_R	GATTGGTACCCATGCCGTCACCCGTACCTG
Luxbox36_F	GATTTCTAGAACTCAGGGGCCCGGTGATGC
Luxbox36_R	GATTGGTACCCACTTGGACCTCGGCGTGGT
Luxbox37_F	GATTTCTAGAGCGGGGATCCTGTGCCGAG
Luxbox37_R	GATTGGTACCCATGGAACGAACCTCCTCAA
Luxbox38_F	GATTTCTAGAGGTTTTCCGGCTATGCACCC
Luxbox38_R	GATTGGTACCCATGCCGCGTTCCTCACTCC
Luxbox40_F	GATTTCTAGAGCGGGCCATGAACGGGTAGA
Luxbox40_R	GATTGGTACCCACGAGGCAGGCCGAATCAG
Luxbox41_F	GATTTCTAGATGGACTGCGGCCTCGGGTTG
Luxbox41_R	GATTGGTACCCATGTTGCCCTCCCGATTGC
Luxbox42_F	GATTTCTAGACGGTTGGAACTCGTGGACGG
Luxbox42_R	GATTGGTACCCATGGTGGCTTCCTTTCTCA
Luxbox44_F	GATTTCTAGATGCCGACCTATAGGCTGAAT
Luxbox44_R	GATTGGTACCCATTGTGATGACCGTCGCCT
Luxbox45_F	GATTTCTAGAGCGGATCGCGTCGTCGGGAA
Luxbox45_R	GATTGGTACCCATCGGACCTTCCGCGGGTG
Luxbox46_F	GATTTCTAGACTCTTCAATACGAAATGGGA
Luxbox46_R	GATTGGTACCCATGGCTGCTCCAAAACACG
Luxbox47_F	GATTTCTAGATTGCGGTGCCGGAACTGGCC
Luxbox47_R	GATTGGTACCCATCCCTCTCTCCTCTGGTT
Luxbox49_F	GATTTCTAGAACCTGGGGGCGGCGATCGAG
Luxbox49_R	GATTGGTACCCATCGGGGCTGTGGGTTCCA
Luxbox50_F	GATTTCTAGAGGTGTTCGGTGTCGGCTCGA
Luxbox50_R	GATTGGTACCCATGGCCGCAGCCTTCCGGC
Luxbox51_F	GATTTCTAGAGCCATATCAACCACACCCGT
Luxbox51_R	GATTGGTACCCATGCGCACGCCTTTGCCGG
Luxbox52_F	GATTTCTAGACAGAGAAACGCCAACAATGT
Luxbox52_R	GATTGGTACCCATGATGAACTCCTTCGCTC
Luxbox53_F	GATTTCTAGACTATGACACCCGTATCTCGC
Luxbox53_R	GATTGGTACCCATAAACCCTCGTGGCCTCC
Luxbox54_F	GATTTCTAGATCATGTGCGGCGAGCACATC
Luxbox54_R	GATTGGTACCCATCGTCCGCCTCCTCTCAT
Luxbox55_F	GATTTCTAGACAGACGGTCGCCCTGTCTGC
Luxbox55_R	GATTGGTACCCATAAGCCCTCGTGGCTCCG
Luxbox56_F	GATTTCTAGACGGTTTGCGGAGGAGGCGAA
Luxbox56_R	GATTGGTACCCATGTGCGCTCCTACTACCC
Luxbox58_F	GATTTCTAGACGTGGACTGCGTCGATGATC
Luxbox58_R	GATTGGTACCCATCGCAGCTCCCCTGCGGG
Luxbox59_F	GATTTCTAGAGGTATCAGGGCGAGGTGCAG
Luxbox59_R	GATTGGTACCCATAGGCGGACTCGTTACGA
Luxbox60_F	GATTTCTAGAAACTGTCCACCTATAAGACC

Luxbox60_R	GATTGGTACCCATGGTAACCCTATGAGTTA
Luxbox61_F	GATTTCTAGACATGTAGGGCCTTGGTTTCA
Luxbox61_R	GATTGGTACCCATAGATCGCACGGGGTGGC
Luxbox62_F	GATTTCTAGACGGAGTGGGTGCTGATCGGC
Luxbox62_R	GATTGGTACCCATGGTGGGCGCTATTCTAA
Luxbox65_F	GATTTCTAGAGCTGCCGCACCCTGTTCATA
Luxbox65_R	GATTGGTACCCATAGGACTCCGAGTGGGCG
Luxbox68_F	GATTTCTAGAGCTCCCGCTTCACACGGTTT
Luxbox68_R	GATTGGTACCGGAGTTCCTCCTTCCGGGCG
Luxbox69_F	GATTTCTAGACGTACCTGGCCGGGCGGCCT
Luxbox69_R	GATTGGTACCGGTGCCTCCTCCTGTGACCA
Luxbox70_F	GATTTCTAGAGGTGCCTCCTCCTGTGACCA
Luxbox70_R	GATTGGTACCCGTACCTGGCCGGGCGGCCT
Luxbox71_F	GATTTCTAGAACGACCAAATCTGCACGCCG
Luxbox71_R	GATTGGTACCAACTCCCCCTCGTGTTGGCG
Luxbox72_F	GATTTCTAGAAGTCGTGCGTCGCCCGTCGT
Luxbox72_R	GATTGGTACCAGGGCCTCTCCTTCCGGTTA

Supplementary Table 2. List of reference *luxI* and *luxR* sequence

Group	Species	Strain	Seq name	Protein accession
luxI reference sequences				
Alpha	Agrobacterium fabrum	C58	Afab_TraI	P33907
Alpha	Agrobacterium tumefaciens		Atum_TraI	AAB95104
Alpha	Agrobacterium vitis		Avit_AvsI	AAY97862
Alpha	Azospirillum lipoferum	TVV3	Alip_AlpI	ABD97989
Alpha	Bradyrhizobium japonicum	USDA 110	Bjap_BjaI	Q89VI2
Alpha	Bradyrhizobium sp.	ORS 278	Brad_BraI	CAL74857.1
Alpha	Mesorhizobium tianshanense		Mtia_MrtI	AAZ32755
Alpha	Rhizobium etli	CNPAF512	Retl_RaiI	AAC38172.1
Alpha	Rhizobium leguminosarum	8401	Rleg_CinI	AAF89990
Alpha	Rhizobium loti (Mesorhizobium loti)	MAFF303099	Mlot_TraI1	BAB52691.1
Alpha	Rhizobium meliloti (Sinorhizobium meliloti)	1021	Smel_SinI	CAC46418.1
Alpha	Rhizobium sp (Sinorhizobium fredii NGR234)	NGR234	Sfre_TraI	P55394.1
Alpha	Rhodobacter capsulatus	SB 1003	Rcap_00329	ADE84094
Alpha	Rhodobacter sphaeroides	2.4.1T	Rsph_CerI	AAC46022
Alpha	Rhodopseudomonas palustris	CGA009	Rpal_RpaI	Q6NCZ6
Alpha	Sinorhizobium meliloti		Smel_TraI	AAR19278
Alpha	Nitrobacter winogradskyi	Nb-255	Nwin_NwiI	ABA03893.1
Beta	Acidovorax citrulli		Acit_AacI	ACP19742.1
Beta	Burkholderia ambifaria		Bamb_BafI	AAK27305.1
Beta	Burkholderia cenocepacia	J2315	Bcen_CciI	CAR54099.1
Beta	Burkholderia cepacia	LMG18943	Bcep_CepI	AAG61128.1
Beta	Burkholderia cepacia	DBO1	Bcep_BviI	AAK35155.1

Beta	Burkholderia glumae	BGR1	Bglu_TofI	AAV52805.1
Beta	Burkholderia kururiensis	M130	Bkur_BraI	CAP91066
Beta	Burkholderia mallei	ATCC 23344	Bmal_BmaI3	AAU45799
Beta	Burkholderia mallei	ATCC 23344	Bmal_BmaI1	AAU46702
Beta	Burkholderia multivorans	ATCC 17616	Bmul_BmuI	AAK71685
Beta	Burkholderia pseudomallei	pp844	Bpse_BpsI	AAM21707.2
Beta	Burkholderia pseudomallei	K96243	Bpse_BpmI	CAH38648
Beta	Burkholderia stabilis	LMG14291	Bsta_CepI	AAG61126.1
Beta	Burkholderia thailandensis	E264	Btha_BtaI	ABC34067.1
Beta	Burkholderia unamae	MTI-641T	Buna_UnaI	CBI71277
Beta	Burkholderia vietnamiensis	R-921	Bvie_CepI	AAG61127.1
Beta	Nitrosospira multiformis		Nmul_NmuI	AGN90587
Beta	Ralstonia solanacearum	AW1	Rsol_SolI	AAC45948.1
Gamma	Acidithiobacillus ferrooxidans		Afer_AfeI	AAZ20805
Gamma	Aeromonas hydrophila	ssu	Ahyd_AhyI2	ABD59318
Gamma	Aeromonas hydrophila	A1	Ahyd_AhyI	CAA61653.1
Gamma	Aeromonas salmonicida	NCIMB 1102	Asal_AsaI	AAB70017.1
Gamma	Aliivibrio fischeri (Vibrio fischeri)		Afis_LuxI	CAA68562
Gamma	Citrobacter rodentium	ICC168	Crod_CroI	CBG89691
Gamma	Edwardsiella tarda		Etar_EdwI	BAD52105
Gamma	Erwinia carotovora (Pectobacterium betavasculorum)	Ecb168	Pbet_EcbI	AAB69645.1
Gamma	Erwinia carotovora (Pectobacterium carotovorum)	SCC3193	Pcar_ExpI	CAA51409.1
Gamma	Erwinia carotovora (Pectobacterium carotovorum)	GS101	Pcar_CarI	CAA52352.1

Gamma	Erwinia chrysanthemi (Dickeya chrysanthemi)	3937	Dchr_ExpI	CAA65306.1
Gamma	Erwinia stewartii (Pantoea stewartii)	SS104	Pste_EsaI	P54656.1
Gamma	Halomonas anticariensis	FP35 = DSM 16096	Hant_HanI	ADN33402
Gamma	Pantoea agglomerans		Pagg_EagI	CAA52353.1
Gamma	Pantoea ananatis		Pan_EanI	BAF69064
Gamma	Pectobacterium atrosepticum	CFBP 6276	Part_expI	CAE45181
Gamma	Pectobacterium carotovorum	SCC1	Pcar_ExpI2	AAR92118.1
Gamma	Pseudomonas aeruginosa	PAO1	Paer_LasI	P33883
Gamma	Pseudomonas aeruginosa	PAO1	Paer_RhlI	P54291
Gamma	Pseudomonas chlororaphis	aureofaciens	Pchl_PhzI	AAC41535
Gamma	Pseudomonas chlororaphis	subsp. aureofaciens	Pchl_CsaI	AAK73191
Gamma	Pseudomonas corrugata	CFBP 5454	Pcor_PcoI	ABP88724.1
Gamma	Pseudomonas fluorescens		Pflu_PcoI	AAT42217
Gamma	Pseudomonas putida		Pput_PpuI	AAM75411
Gamma	Pseudomonas syringae	CFBP 10912-9	Psyr_PsmI	AAK15016.1
Gamma	Pseudomonas syringae	tabaci	Psyr_PsyI	P52990
Gamma	Pseudomonas syringae	syringae	Psyr_AhlI	CAD98865
Gamma	Serratia liquefaciens	MG1	Sliq_SwrI	AAB18141.1
Gamma	Serratia marcescens		Smar_SpnI	AAN52498.1
Gamma	Serratia plymuthica	48	Sply_SplI	AAW27921.1
Gamma	Serratia proteamaculans	B5a	Spro_SprI	AAK76733.1
Gamma	Serratia sp	ATCC 39006	Serr_SmaI	CAB92553.1
Gamma	Vibrio anguillarum	NB10	Vang_VanI	AAC45212.1
Gamma	Vibrio fischeri	ES114	Vfis_LuxI1	AAA27543.1
Gamma	Yersinia enterocolitica	NCTC 10460	Yent_YenI	CAA53693.1
Gamma	Yersinia pseudotuberculosis		Ypse_YtbI	AAC28704.2

Gamma	Yersinia pseudotuberculosis		Ypse_YpsI	AAD40486.1
Gamma	Yersinia ruckeri		Yruc_YukI	O87970
	uncultured bacterium		Ubac_AubI	BAM45368
	uncultured bacterium		Ubac_AusI	BAM45374
	uncultured bacterium (QS10-1-4)		QS10-1-4	ACH69667.1
	uncultured bacterium (QS10-2-3)		QS10-2-3	ACH69672.1
	uncultured bacterium (QS6-1-5)		QS6-1-5	ACH69662.1
	uncultured proteobacterium	QS1	QS1	AAT90822.1
	uncultured alphaproteobacterium		Ubac_TswI	KP092521
	Nitrospira japonica	NJ1		WP_080886260.1
	Candidatus Nitrospira nitrificans			CUS35775.1
	Nitrospira moscoviensis	M1		WP_053378364.1
	Candidatus Nitrospira inopinata			WP_062484320.1
luxR reference sequences				
Alpha	Agrobacterium fabrum	c58	Afab_TraR	P54294
Alpha	Agrobacterium tumefaciens		Atum_TraR	AAC28121
Alpha	Agrobacterium vitis		Avit_AvsR	AAY97861.1
Alpha	Azospirillum lipoferum		Alip_AlpR	ABD97988
Alpha	Bradyrhizobium japonicum	USDA 110	Bjap_BjaR	NP_767702.1
Alpha	Bradyrhizobium sp.	ORS 278	Brad_BraR	CAL74858
Alpha	Mesorhizobium tianshanense		Mtia_MrtR	AAZ32754.1
Alpha	Rhizobium etli	CNPAF512	Retl_RaiR	AAC38173.1
Alpha	Rhizobium leguminosarum	8401	Rleg_RaiR	CAD20930.1

Alpha	Rhizobium leguminosarum	8401	Rleg_CinR	AAF89989
Alpha	Rhizobium leguminosarum	bv. viciae	Rleg_RhiR	AAA26360
Alpha	Rhizobium sp (Sinorhizobium fredii NGR234)	NGR234	Sfre_TraR	AAB91637.1
Alpha	Rhodobacter capsulatus	SB 1003	Rcap_00328	ADE84094
Alpha	Rhodobacter sphaeroides	2.4.1T	Rsph_CerR	AAC46021.1
Alpha	Rhodopseudomonas palustris	CGA009	Rpal_RpaR	CAE25765.1
Alpha	Sinorhizobium meliloti	1021	Smel_SinR	CAC46417
Alpha	Sinorhizobium meliloti		Smel_TraR	AAR19282
Alpha	Nitrobacter winogradskyi	Nb-255	Nwin_NwiR	ABA03894.1
Beta	Acidovorax citrulli		Acit_AacR	ACP19743
Beta	Burkholderia ambifaria		Bamb_BafR	AAK27306.1
Beta	Burkholderia cenocepacia	J2315	Bcen_CciR	CAR54100
Beta	Burkholderia cepacia	DBO1	Bcep_BviR	AAK35156.1
Beta	Burkholderia cepacia	LMG18943	Bcep_CepR	AAD12726.1
Beta	Burkholderia glumae	BGR1	Bglu_TofR	AAV52804
Beta	Burkholderia kururiensis	M130	Bkur_BraR	CAP91064
Beta	Burkholderia mallei	ATCC 23344	Bmal_BmaR3	AAU45800
Beta	Burkholderia multivorans	LMG16660	Bmul_CepR	AAG61130.1
Beta	Burkholderia multivorans	ATCC 17616	Bmul_BmuR	AAK50054.2
Beta	Burkholderia pseudomallei		Bpse_BpsR	AAS90557
Beta	Burkholderia pseudomallei	K96243	Bpse_BpmR	CAH38644
Beta	Burkholderia stabilis	LMG14291	Bsta_CepR	AAK70350.1
Beta	Burkholderia thailandensis	E264	Btha_BtaR	ABC34774
Beta	Burkholderia unamae		Buna_UnaR	CBI71275.1

Beta	Burkholderia vietnamiensis	C2822	Bvie_BviR	ABK32016.1
Beta	Burkholderia vietnamiensis	PC259	Bvie_CepR	AAK70352.1
Beta	Nitrosospira multiformis		Nmul_NmuR	AHB23331.1
Beta	Ralstonia solanacearum	AW1	Rsol_SolR	AAC45947.1
Gamma	Acidithiobacillus ferrooxidans		Afer_AfeR	AAV53702.2
Gamma	Aeromonas hydrophila	A1	Ahyd_AhyR	CAA61654.1
Gamma	Aeromonas hydrophila		Ahyd_AhyR2	ABD59317
Gamma	Aeromonas salmonicida	NCIMB 1102	Asal_AsaR	AAB70018.1
Gamma	Aliivibrio fischeri		Afis_LuxR	CAA68561
Gamma	Citrobacter rodentium	ICC168	Crod_CroR	CBG89690
Gamma	Dickeya chrysanthemi		Dchr_EchR	AAA86840
Gamma	Edwardsiella tarda		Etar_EdwR	BAD52106
Gamma	Erwinia carotovora (Pectobacterium carotovorum)	GS101	Pcar_CarR	AAC45995.1
Gamma	Erwinia carotovora (Pectobacterium carotovorum)	SCC3193	Pcar_ExpR	CAA56646.1
Gamma	Erwinia carotovora (Sinorhizobium fredii NGR234)	Ecb168	Pbet_EcbR	AAB69646.1
Gamma	Erwinia chrysanthemi	3937	Echr_ExpR	CAA65307.1
Gamma	Erwinia stewartii (Pantoea stewartii)	DC283	Pste_EsaR	AAA82097
Gamma	Escherichia coli K12	K12 MG1655	Ecol_SdiA	P07026.2
Gamma	Halomonas anticariensis	FP35 = DSM 16096	Hant_HanR	ADN33401.1
Gamma	Pantoea ananatis		Pan_EanR	BAF69065
Gamma	Pseudomonas aeruginosa	PA01	Paer_LasR	NP_250121.1
Gamma	Pseudomonas aeruginosa	PA01	Paer_PhzR	AAF01471.1
Gamma	Pseudomonas aeruginosa	PA01	Paer_RhlR	NP_252167.1
Gamma	Pseudomonas chlororaphis	PCL1391	Pchl_PhzR	AAF17494.1

Gamma	Pseudomonas chlororaphis	aureofaciens	Pchl_PhzR2	AAA21841
Gamma	Pseudomonas corrugata	CFBP 5454	Pcor_PcoR	ABP88722.1
Gamma	Pseudomonas fluorescens	BL915	Pflu_AfmR	AAF61717.1
Gamma	Pseudomonas fluorescens		Pflu_PcoR	AAT42219.1
Gamma	Pseudomonas fluorescens	BL915	Pflu_RhlR	AAF61719.1
Gamma	Pseudomonas putida		Pput_PpuR	AAM75413.1
Gamma	Pseudomonas syringae	CFBP 10912-9	Psyr_PsmR	AAK15017.1
Gamma	Pseudomonas syringae	pv. tabaci	Psyr_PsyR	AAF26601.1
Gamma	Pseudomonas syringae	syringae	Psyr_AhlR	CAD97431
Gamma	Serratia liquefaciens	MG1	Sliq_SwrR	AAO38761.1
Gamma	Serratia marcescens	ATCC 39006	Smar_CarR	AAC38168.1
Gamma	Serratia marcescens		Smar_SpnR	AAN52499.1
Gamma	Serratia plymuthica		Sply_SplR	AAW27922
Gamma	Serratia proteamaculans	B5a	Spro_SprR	AAK76734.1
Gamma	Serratia sp	ATCC 39006	Serr_SmaR	CAB92554.1
Gamma	Vibrio anguillarum	NB10	Vang_VanR	AAC45213.1
Gamma	Yersinia enterocolitica	NCTC 10460	Yent_YenR	CAA53694.1
Gamma	Yersinia pseudotuberculosis		Ypse_YpsR	AAD40485.1
Gamma	Yersinia pseudotuberculosis		Ypse_YtbR	AAC28703.2
Gamma	Yersinia ruckeri		Yruc_YruR	AAG17669.1
Gamma	Yersinia ruckeri		Yruc_YukR	O87971
	Uncultured bacterium		Ubac_AubR	BAM45367.1
	Uncultured bacterium		Ubac_AusR	BAM45373.1
	uncultured bacterium (QS10-1-3)		QS10-1-3	ACH69668.1
	uncultured bacterium (QS10-2-2)		QS10-2-2	ACH69671.1
	uncultured bacterium (QS6-1-6)		QS6-1-6	ACH69663.1

	uncultured proteobacterium	QS1	QS1	AAT90827.1
	uncultured alphaproteobacterium		Ubac_TswR	KP092522
	Nitrospira japonica	NJ1		WP_080886261.1
	Candidatus Nitrospira nitrificans			CUS35777.1
	Nitrospira moscoviensis	M1		ALA56953.1
	Candidatus Nitrospira inopinata			WP_062484322.1

Supplementary Table 3. List of reference *lux* box sequences

**Gene**	***lux* box sequences**
aubIbox	ACCTGGCGGTTCCGCCAGGT
ausI	AACTACCAGATCTGATAGCT
rhlI	CCCTACCAGATCTGGCAGGT
solI	CCCTGTCAATCCTGACAGTT
luxI	ACCTGTAGGATCGTACAGGT
lasI	ACCTGCGAGAACTGGCAGGT
afeI	AGCTGTCAACCTTGACAGCT
cepI	CCCTGTAAGAGTTACCAGTT
rpaI1	ACCTGTCCGATCGGACAGTA
rpaI2	CACTGTTCCCGCCTGCAGAC
psmI	ACCTGTTCCTAGGTACAGTA
esaI	ACCTGCACTATAGTACAGGC
phzA	ACCTACCAGATCTTGTAGTT
vanI	AACTGTTCGATCGAACAGGT
spnR	ACCTGACCGAAAGGTCAGGT
recA	TACTGTATGACCATACAGTA
esaR	ACCTGCACTATAGTACAGTA
xccR	ACCTTGGCAATTTGCCAGTT
oryR	ACCTGTGAGATTTGCCAGTT
lecA	TCCTGCATGAATTGGTAGGC
vioA	CCCTGACCCTTGGAACAGTA
psoA	TTCTGCAGGCTTCTACAGGT
ppuI	ACCTCCCAAATTAGGTAGGA
ppuA	ACCTCCCTGTTCTGGGAGGT
luxI_ATCC	ACCTGTAGGATCGTACAGGT
qsrP__MJ1	ACCTGTAATAAGTTACAGGA
qsrP_ES114	ACCTGTAATAAACGACAGGA
aidA	ACCTGTTTACTTTTACAGCT
phzM	AACTACAAGATCTGGTAGGT
rsaL	AACTAGCAAATGAGATAGAT
rhlAB	TCCTGTGAAATCTGGCAGTT
braI	ACCTATCCAGGTAGGTAGGT
xenI	ACCTATCCAGGTAGGTAGGT
phyI	ACCTATCCAGGTAGGTAGGT
unaI	ACCTACCTATCTAGATAGGT
luxI_MJ1	ACCTGTAGGATCGTACAGGT
bmaI	CCCTGTAAGGGTTAACAGTT
pipI	ACCTGAACGCCCGTTCTGCG
phzI	CACTACAAGATCTGGTAGTT
PA1897 (qsc102)	ACCTGCCCGGAAGGGCAGGT

qsc104	AACTACCAGTTCTGGTAGGT
qsc117	CACTGCCAGATCTGGCAGTT
qsc126	ACCTAACAGATTTGTAAGTT
qsc128	ACCTACCAGAATTGGCAGGG
qsc131	ACCTACCAGATCTTGTAGGT
qsc132A&B	CCCTACGCGGCCTTGGAGCC
lasB, op1	ACCTGCCAGTTCTGGCAGGT
hcnA	ACCTACCAGAATTGGCAGGG
bpsR	CGCTGTCATACTTGCTAGGT
bpsI	CCCTGTAAGGGTTAACAGTT
luxI_ES114	AGCTGTAGGATGGTACAGGT
luxI_H905	GGCTGTAGGATAGTACAGGT
luxI_EM30	GCCTGTAGGATCGTACAGGT
PA1131-rhlC	ACCTGTGAATTCCGGTAGTT

Supplementary Table 4. List of Nspbox identified and their associated genes

Scaffold id	Nspbox	matched sequence	p-value	q-value	RAST identfiication	JGI identification
scaffold37017_7.9_0.618_130596_	Nspbox 1	ACCTGGCGGTTCCGCCAGGT	7.59E-07	0.0185	diguanylate cyclase/phosphodiesterase (GGDEF & EAL domains) with PAS/PAC sensor(s)	hypothetical protein
scaffold37017_7.9_0.618_130596_	Nspbox 2	ACCTGGCGGAACCGCCAGGT	8.51E-07	0.0185	N-acyl-L-homoserine lactone synthetase	N-acyl-L-homoserine lactone synthetase
scaffold37017_7.9_0.618_130596_	Nspbox68	ACCTACCCCGGTGGGTAGGT	3.55E-06	0.045	N.A.	hypothetical protein
scaffold37017_7.9_0.618_130596_		AACTGTCCGGCCTTGAAGGT	4.15E-06	0.045	N.A.	hypothetical protein
scaffold87880_8.1_0.608_101431_	Nspbox 3	CCCTGTGAACCCCGCCAGGT	1.79E-05	0.589	DNA polymerase III subunits gamma and tau	DNA polymerase-3 subunit gamma/tau
scaffold87880_8.1_0.608_101431_		ACCTATCGATTTTCCTAGTT	3.78E-05	0.589	N.A.	hypothetical protein
scaffold87880_8.1_0.608_101431_		AACTAGGAAAATCGATAGGT	4.89E-05	0.589	N.A.	methylenetetrahydrofolate--tRNA-(uracil-5-)-methyltransferase
scaffold147498_8.2_0.619_105907_		GCCTGCACAACCGTCCAGGT	2.69E-06	0.059	N.A.	hypothetical protein
scaffold147498_8.2_0.619_105907_	Nspbox 4	TCCTGCCCGAACGGGGAGGG	3.03E-06	0.059	RNA polymerase factor sigma-70	RNA polymerase sigma-70 factor, ECF subfamily
scaffold147498_8.2_0.619_105907_		CCCTACACGATGGCGCAGAT	4.46E-05	0.482	N.A.	hypothetical protein

scaffold147498_8.2_0.619_105907_	Nspbox 5.1	CACTGCCACACCGTACTGGT	4.95E-05	0.482	Vegetative cell wall protein gp1 precursor (Hydroxyproline-rich glycoprotein 1)	Tetratricopeptide repeat-containing protein;
scaffold147498_8.2_0.619_105907_		ACCTACAACATCGAGAAGGC	7.61E-05	0.591	N.A.	hypothetical protein
scaffold147498_8.2_0.619_105907_		TCCTGGGGGATCAATCAGGT	9.10E-05	0.591	N.A.	hypothetical protein
scaffold66391_7.9_0.603_88979_		ACCTCTATCGTCTCACAGGT	2.14E-05	0.7	N.A.	Uncharacterized membrane protein YciS, DUF1049 family
scaffold66391_7.9_0.603_88979_		ACCTTTCGACCCAGTCAGGT	6.99E-05	1	N.A.	DNA-binding transcriptional regulator, MerR family
scaffold82577_7.2_0.607_78587_	Nspbox 6	AGCTGCCGATTTCTCCAGGA	4.83E-05	0.488	Branched-chain amino acid aminotransferase (EC 2.6.1.42)	branched-chain amino acid aminotransferase
scaffold82577_7.2_0.607_78587_		GCCTGTCTCATGACACAGGT	4.83E-05	0.488	N.A.	hypothetical protein
scaffold82577_7.2_0.607_78587_	Nspbox 7	TCCTGGAGAAATCGGCAGCT	5.13E-05	0.488	LSU ribosomal protein L27p	large subunit ribosomal protein L27
scaffold82577_7.2_0.607_78587_		ACCTCTCGCAGCCGTCAGGG	7.79E-05	0.556	N.A.	Signal transduction histidine kinase regulating C4-dicarboxylate transport system
scaffold48969_8.2_0.627_127797_	Nspbox 8	GCCTACCGACGCTGGCAGGC	2.63E-05	0.954	N.A.	Glycosyltransferase
scaffold48969_8.2_0.627_127797_		CCCTGCCGACGCTGAAAGGG	5.91E-05	0.954	N.A.	peptide chain release factor 3
scaffold48969_8.2_0.627_127797_		CCCTGCCGACGCTGAAAGGG	5.91E-05	0.954	N.A.	hypothetical protein

scaffold48969_8.2_0.627_127797_	Nspbox 9	GGCTGTTCGGTTCCGCAGGT	8.33E-05	0.954	RNA polymerase sigma-70 factor, ECF subfamily	RNA polymerase sigma-70 factor, ECF subfamily
scaffold48969_8.2_0.627_127797_	Nspbox 10	ATCTGCCCGTCGGAGCAGGA	9.61E-05	0.954	NAD(FAD)-utilizing dehydrogenases	NAD(FAD)-utilizing dehydrogenases
scaffold66835_8.3_0.613_86824_		GCCTGGCGGGACCGGCAGTA	7.07E-05	1	N.A.	BT1 family protein
scaffold31953_7.9_0.612_73984_		AACTTCCGGATCGGGCAGGT	4.59E-07	0.0128	N.A.	hypothetical protein
scaffold64694_7.6_0.608_67854_		TCCTGCTCGGTTCGGTAGGG	1.52E-05	0.428	N.A.	ADP-ribose pyrophosphatase YjhB, NUDIX family
scaffold42434_7.8_0.612_62990_	Nspbox 11	ACCTGCCGGCGCGGCGAGGG	2.40E-05	0.631	Cell division protein FtsZ	cell division protein FtsZ
scaffold30397_8.1_0.623_52564_		GCCTATCGCATCATGCAGGA	2.29E-05	0.456	N.A.	two-component system, NtrC family, response regulator AtoC
scaffold68691_7.5_0.589_55102_		ACCTTCCCACTTTCGTAGGT	2.06E-05	0.225	N.A.	hypothetical protein
scaffold68691_7.5_0.589_55102_	Nspbox 12	ACCTACGAAAGTGGGAAGGT	2.63E-05	0.225	Soluble lytic murein transglycosylase	soluble lytic murein transglycosylase
scaffold68691_7.5_0.589_55102_		TACTGCCGACCCTGTCAGGG	4.23E-05	0.225	N.A.	Right-handed beta helix region
scaffold68691_7.5_0.589_55102_	Nspbox 13	CCCTGACAGGGTCGGCAGTA	4.38E-05	0.225	Hypothetical protein	tol-pal system protein YbgF
scaffold68691_7.5_0.589_55102_	Nspbox 14	TCCTGCACAAGGCCACAGTT	5.74E-05	0.235	diguanylate cyclase/phosphodiesterase (GGDEF & EAL domains) with PAS/PAC sensor(s)	diguanylate cyclase (GGDEF) domain-containing protein
scaffold44735_7.5_0.623_74690_		CACTCCAAGGTGGTGCAGGT	2.17E-05	0.641	N.A.	hypothetical protein

scaffold44735_7.5_0.623_74690_		ACCTGTCAGCTCTGCCCGGA	9.51E-05	1	N.A.	hypothetical protein
scaffold38022_7.3_0.611_85444_	Nspbox 15	ACCTGGGCCGTCGGCCAGGT	1.05E-05	0.34	membrane protein, putative	Glutaredoxin
scaffold38022_7.3_0.611_85444_		TCCTATCGGGTTTGCCAGGC	1.72E-05	0.34	N.A.	diaminopimelate dehydrogenase
scaffold38022_7.3_0.611_85444_	Nspbox 16	GCCAGACAGATCGGGCAGGG	3.17E-05	0.417	Cytochrome c, class I	Cytochrome C oxidase, cbb3-type, subunit III
scaffold38022_7.3_0.611_85444_		ACCTTCCGGCGATTTCAGGT	8.01E-05	0.711	N.A.	hypothetical protein
scaffold38022_7.3_0.611_85444_	Nspbox 17	ACCTGAAATCGCCGGAAGGT	9.00E-05	0.711	18K peptidoglycan-associated outer membrane lipoprotein; Peptidoglycan-associated lipoprotein precursor; Outer membrane protein P6; OmpA/MotB precursor	peptidoglycan-associated lipoprotein
scaffold103094_7.2_0.616_46026_		ACCTGACCGGGCGAACAGGC	1.51E-05	0.272	N.A.	hypothetical protein
scaffold103094_7.2_0.616_46026_		ACCTGCGAGGACCATCAGGA	6.53E-05	0.436	N.A.	ATP-dependent Clp protease ATP-binding subunit ClpB
scaffold103094_7.2_0.616_46026_	Nspbox 18	ACCTGCACGTCTTTGGTGGT	8.76E-05	0.436	GLUCOSE-FRUCTOSE OXIDOREDUCTASE (EC 1.1.99.28)	Predicted dehydrogenase
scaffold103094_7.2_0.616_46026_		ACCTGCGAAGAGGAGCAGGC	9.67E-05	1	MG(2+) CHELATASE FAMILY PROTEIN / ComM-related protein	N.A.
scaffold56835_7.9_0.603_43269_	Nspbox 19	CACTATCTGGATGGGCAGGT	6.57E-06	0.116	N.A.	exodeoxyribonuclease VII large subunit

scaffold56835_7.9_0.603_43269_		TCCTGCGCGGCCCGATAGGC	5.16E-05	0.324	N.A.	para-aminobenzoate synthetase component 1
scaffold56835_7.9_0.603_43269_	Nspbox 20	GCCTATCGGGCCGCGCAGGA	5.51E-05	0.324	ATP synthase F0 sector subunit a	F-type H+-transporting ATPase subunit a
scaffold95532_7.2_0.617_48473_		CCCTGTATGCTGCTGGAGGT	5.38E-05	0.508	N.A.	Protein of unknown function
scaffold95532_7.2_0.617_48473_	Nspbox 21	ACCTCCAGCAGCATACAGGG	5.95E-05	0.508	Probable glucarate transporter	MFS transporter, ACS family, glucarate transporter
scaffold95532_7.2_0.617_48473_		ACCTGCTCGATCGCGCCGGT	9.20E-05	0.525	N.A.	Tetratricopeptide repeat-containing protein
scaffold101378_7.3_0.628_47338_		CCCTGTATGCCGTGGCAGGT	3.29E-06	0.0549	N.A.	hypothetical protein
scaffold101378_7.3_0.628_47338_		ACCTATCAATCCGAGGAGGA	5.51E-05	0.459	N.A.	hypothetical protein
scaffold101378_7.3_0.628_47338_	Nspbox 22	ACCTACGACCAGCGGCAGTT	8.52E-05	0.473	Glucose-1-phosphate thymidylyltransferase (EC 2.7.7.24)	glucose-1-phosphate thymidylyltransferase
scaffold7514_7.9_0.613_50599_		AACTGTACACTCGTCCAGTG	1.31E-05	0.114	N.A.	hypothetical protein
scaffold7514_7.9_0.613_50599_	Nspbox 23	CACTGGACGAGTGTACAGTT	1.37E-05	0.114	Transcriptional regulator	Helix-turn-helix domain-containing protein
scaffold26245_7.1_0.594_47793_	Nspbox 24	AGCTGCCCGCATTCGCAGCG	5.61E-05	0.353	Thiamin biosynthesis lipoprotein ApbE	thiamine biosynthesis lipoprotein
scaffold26245_7.1_0.594_47793_		CGCTGCGAATGCGGGCAGCT	5.88E-05	0.353	N.A.	hypothetical protein
scaffold26245_7.1_0.594_47793_	Nspbox 25	AACTACCTGGCCGAGCAGAT	7.24E-05	0.353	Bacterioferritin	bacterioferritin
scaffold26245_7.1_0.594_47793_		AACTGCCCGACCTGGAAGAC	8.38E-05	0.353	N.A.	PAS domain S-box-containing protein

scaffold48023_7.0_0.593_38397_	Nspbox 26	ACCTGGTCAATAAGACAGGC	2.70E-05	0.373	Positive regulator of CheA protein activity (CheW)	purine-binding chemotaxis protein CheW
scaffold8220_6.9_0.584_34760_		ACCTATGGTTCTTCGCAGGT	6.41E-05	0.47	N.A.	preprotein translocase subunit YajC
scaffold8220_6.9_0.584_34760_		ACCTGTTAGTTCGGATTGTA	8.24E-05	0.47	N.A.	heptosyltransferase-2
scaffold65686_7.6_0.613_42777_	Nspbox 27	AACTCCTCGGTCGCGCAGTT	4.55E-05	0.315	Signal transduction histidine kinase CheA (EC 2.7.3.-)	chemosensory pili system protein ChpA (sensor histidine kinase/response regulator)
scaffold65686_7.6_0.613_42777_	Nspbox 28	CACTACTCGGCCCTGCAGGG	6.72E-05	0.315	N.A.	Lipopolysaccharide biosynthesis regulator YciM, contains six TPR domains and a predicted metal-binding C-terminal domain
scaffold65686_7.6_0.613_42777_	Nspbox 29	CCCTGCAGGGCCGAGTAGTG	6.95E-05	0.315	N.A.	lipopolysaccharide export system permease protein
scaffold102524_8.2_0.602_27400_	Nspbox 30	TGCTGTCAGACGTGACAGTC	2.73E-05	0.294	Ferredoxin--sulfite reductase	sulfite reductase (ferredoxin)
scaffold176833_14.5_0.595_35983_	Nspbox 31	TACTGTCTGCTCTGGCAGGT	2.14E-07	0.0023	Glycosyltransferase	Glycosyltransferase involved in cell wall bisynthesis
scaffold176833_14.5_0.595_35983_	Nspbox 32	TCCTGTGAAACCATGCAGCT	2.07E-05	0.111	Glycosyltransferase	Glycosyl transferases group 1
scaffold162628_7.3_0.597_24950_		AACAGCATGACCTTGCAGGA	5.71E-05	0.632	N.A.	cysteine desulfurase IscS

scaffold79_7.5_0.615_30129_	Nspbox 33	TCCTGCCTGAACTGAGAGTT	4.25E-06	0.0457	ATP-dependent Clp protease ATP-binding subunit ClpX	ATP-dependent Clp protease ATP-binding subunit ClpX
scaffold79_7.5_0.615_30129_	Nspbox 34	GCCTTCAAGAACATGCAGGA	6.56E-05	0.353	N.A.	Peptidoglycan-synthase activator LpoB; NIDE2049
scaffold28140_7.5_0.603_24203_		GCCTGTGTCGCCTGACAGGG	7.70E-05	0.769	N.A.	Radical SAM superfamily enzyme YgiQ, UPF0313 family
scaffold30796_7.7_0.618_24509_		AACTGCAATTTCGCGCAGCC	6.16E-05	0.267	N.A.	PAS domain S-box-containing protein
scaffold30796_7.7_0.618_24509_	Nspbox 35	ACCTTCCAGGTGCCCCAGGT	6.95E-05	0.267	two component transcriptional regulator, LuxR family	DNA-binding response regulator, NarL/FixJ family, contains REC and HTH domains; two component transcriptional regulator, LuxR family
scaffold92675_7.8_0.625_29064_		CACTGCAACCTCGGTCAGTG	5.64E-05	0.349	N.A.	YD repeat-containing protein
scaffold92675_7.8_0.625_29064_		ACCTTTACGATCGTGGAGCG	7.28E-05	0.349	N.A.	membrane fusion protein, cobalt-zinc-cadmium efflux system
scaffold67668_7.4_0.593_23384_	Nspbox 36	ACCTCCTCAACTGTGCAGGG	2.70E-05	0.114	Membrane-bound lytic murein transglycosylase D precursor	membrane-bound lytic murein transglycosylase D
scaffold67668_7.4_0.593_23384_	Nspbox 37	CCCTGCACAGTTGAGGAGGT	2.82E-05	0.114	Serine--glyoxylate aminotransferase	aspartate aminotransferase; sgaA
scaffold56188_7.3_0.608_33128_		GCCTGGTCCAATGGACAGTT	7.07E-05	0.258	N.A.	Hypothetitcal protein
scaffold56188_7.3_0.608_33128_		GACTGTCCTTCGTGGCAGGA	7.66E-05	0.258	N.A.	Hypothetitcal protein

scaffold56188_7.3_0.608_33128_		TCCTGCCACGAAGGACAGTC	7.70E-05	0.258	N.A.	Hypothetitcal protein
scaffold73194_6.8_0.599_33749_	Nspbox 38	CCCTCGCCGGTCTGGCAGGC	1.44E-05	0.217	Urea ABC transporter, ATPase protein UrtD	urea transport system ATP-binding protein
scaffold14848_7.3_0.614_38099_	NA	ACCTGCCAGCTTCTCCTGGA	7.44E-05	0.998	Poly(glycerol-phosphate) alpha-glucosyltransferase (EC 2.4.1.52)	N.A.
scaffold14848_7.3_0.614_38099_		AGCTGCCATGGCGTGTAGTC	7.61E-05	0.304	N.A.	hypothetical protein
scaffold14848_7.3_0.614_38099_	Nspbox 39	GACTACACGCCATGGCAGCT	7.97E-05	0.304	Long-chain fatty acid transport protein	long-chain fatty acid transport protein
scaffold174546_16.7_0.600_27128_	Nspbox 40	GCCTGGAGGAATCGGCAGCT	3.97E-05	0.255	K30 capsule biosynthesis cluster, partial sequence	Capsule assembly protein Wzi
scaffold174546_16.7_0.600_27128_		CCCTCGACGTATTGCCAGGT	6.64E-05	0.255	N.A.	hypothetical protein
scaffold98569_6.8_0.617_18799_		ACCTGGCCGATCGTGAAGCC	3.64E-05	0.252	N.A.	glycyl-tRNA synthetase beta chain
scaffold98569_6.8_0.617_18799_		CACTATACGTTGAGCCAGTT	8.05E-05	0.278	N.A.	sulfate transport system substrate-binding protein
scaffold111638_7.3_0.585_19797_		TACTGTCCGAATTGCCAGCG	2.66E-05	0.121	N.A.	ATP-citrate lyase beta-subunit; aclB
scaffold41520_6.9_0.605_14879_	Nspbox 41	GACTACTGGAACCGGCAGTT	4.55E-05	0.284	OsmC/Ohr family protein	peroxiredoxin, SACOL1771 subfamily
scaffold41520_6.9_0.605_14879_	Nspbox 42	TCCTGTACAACTTGTCTGGT	8.71E-05	0.284	N.A.	Cytochrome c553
scaffold169997_7.7_0.595_14562_		CGCTGTCCGGTTCGGAAGGT	3.36E-05	0.116	N.A.	hypothetical protein

scaffold169997_7.7_0.595_14562_	Nspbox 43	ACCTTCCGAACCGGACAGCG	3.55E-05	0.116	ATP-dependent DNA helicase UvrD/PcrA, proteobacterial paralog	DNA helicase-2 / ATP-dependent DNA helicase PcrA
scaffold175053_13.5_0.560_12102_	Nspbox 44	ACCTCCATAGAATTGCAGGT	3.03E-05	0.111	Uncharacterized protein ImpJ/VasE	type VI secretion system protein ImpJ
scaffold35534_8.4_0.593_17546_		AGCTGTATGGGTTGAAAGGT	5.35E-05	0.246	N.A.	Methyltransferase domain-containing protein
scaffold132765_6.3_0.604_10581_		ACCTGCCCATTCTGTCAGGC	5.78E-07	0.0017	N.A.	Ankyrin repeat-containing protein
scaffold174996_7.1_0.618_10136_	Nspbox 45	AGCTGTTCAACCGTCCAGGT	7.95E-06	0.0114	glycosyl transferase, family 2	dolichyl-phosphate beta-glucosyltransferase
scaffold174996_7.1_0.618_10136_		ACCTGGACGGTTGAACAGCT	8.50E-06	0.0114	N.A.	hypothetical protein
scaffold174996_7.1_0.618_10136_		CACTGCCACAGCGTCGAGGT	5.25E-05	0.0443	N.A.	hypothetical protein
scaffold174996_7.1_0.618_10136_		CCCTCCACGCATCGACAGCT	7.40E-05	0.0443	N.A.	dolichyl-phosphate beta-glucosyltransferase
scaffold174996_7.1_0.618_10136_		AGCTGTCGATGCGTGGAGGG	8.24E-05	0.0443	N.A.	hypothetical protein
scaffold70518_5.5_0.617_10666_		ACCTGGCCGGGCGGCCTGGT	4.60E-05	0.0798	N.A.	Hypothetical protein
scaffold70518_5.5_0.617_10666_	Nspbox69	ACCTGGCCGGGCGGCCTGGT	4.60E-05	0.0798	N.A.	Hypothetical protein
scaffold70518_5.5_0.617_10666_	Nspbox70	ACCAGGCCGCCCGGCCAGGT	4.77E-05	0.0798	N.A.	Hypothetical protein
scaffold70518_5.5_0.617_10666_		ACCTATCAGTCATTGCTGGA	9.61E-05	0.121	N.A.	Hypothetical protein
scaffold9556_5.2_0.605_10727_	Nspbox 46	GCCTGGTACTTCTGACAGGC	4.98E-05	0.16	DNA-3-methyladenine glycosylase II (EC 3.2.2.21)	DNA-3-methyladenine glycosylase

scaffold177594_8.0_0.604_11943_		TCCTACGAGGCCGTGCAGCG	7.88E-05	0.423	N.A.	chlorite dismutase
scaffold177969_7.9_0.625_14543_		ACCTCGACGACGTGACAGCG	9.10E-05	0.467	N.A.	16S rRNA (guanine(966)-N(2))-methyltransferase RsmD
scaffold102971_5.8_0.597_10059_		ACCTATACCCTGTGACAGTC	3.23E-05	0.117	N.A.	D-alanyl-D-alanine carboxypeptidase (penicillin-binding protein 5/6); NIDE0434
scaffold47892_5.6_0.597_9662_		ACCTGGCAACCCTGGGAGTT	8.63E-06	0.0198	N.A.	Response regulator receiver domain-containing protein
scaffold47892_5.6_0.597_9662_		ACCTCGCTGGTACGGCAGGA	8.10E-05	0.093	N.A.	Response regulator receiver domain-containing protein
scaffold57321_6.1_0.594_10417_		CCCTGCTATCTGTTGCAGGA	3.76E-05	0.0758	N.A.	hypothetical protein
scaffold57321_6.1_0.594_10417_	Nspbox 47	TCCTGAAAAGACCGATAGTT	4.38E-05	0.0758	Molybdenum cofactor biosynthesis protein MoaA	hopanoid biosynthesis associated radical SAM protein HpnH
scaffold65098_6.7_0.588_11234_		GCCTATCTACGCCGACAGGG	4.52E-05	0.208	N.A.	PAS domain S-box-containing protein
scaffold114626_6.0_0.623_8921_		GCCTGCACGATCTGCCAGCC	8.01E-06	0.023	N.A.	YggS family pyridoxal phosphate enzyme
scaffold92604_6.8_0.605_8759_	Nspbox 48	TCCTGCCGGCCGTGACAGTA	2.90E-05	0.0377	Lipolytic enzyme, G-D-S-L precursor	Lysophospholipase L1
scaffold92604_6.8_0.605_8759_	Nspbox 49	TACTGTCACGGCCGGCAGGA	3.09E-05	0.0377	PBS lyase HEAT domain protein repeat-containing protein	hypothetical protein
scaffold105453_6.9_0.617_9874_		ACCTACAAGTTGGACGAGGT	6.16E-05	0.189	N.A.	Protein of unknown function (DUF3047)

scaffold46119_6.9_0.614_9228_	NA	ACCTGCTCCGTCCAGTAGTG	9.46E-05	1	GTP-binding protein EngA	N.A.
scaffold138987_7.0_0.594_7804_		ACCTGTTTGCTCGAGAAGTT	7.92E-05	0.182	N.A.	phenylalanyl-tRNA synthetase beta subunit (EC 6.1.1.20)
scaffold31174_4.9_0.588_7480_		GACTCCGATTTCGGACAGGT	7.36E-05	0.123	N.A.	membrane fusion protein, multidrug efflux system
scaffold105484_6.4_0.596_8074_		GCCTATCAACCCGGACAGTT	4.39E-06	0.0118	N.A.	CDP-diacylglycerol--serine O-phosphatidyltransferase (EC 2.7.8.8)
scaffold105484_6.4_0.596_8074_		ATCTGTTGGGTGGGGCAGGG	5.84E-05	0.0783	N.A.	CDP-diacylglycerol--serine O-phosphatidyltransferase (EC 2.7.8.8)
scaffold23163_6.4_0.605_8521_	Nspbox 50	GCCTATTGGATGGTGCAGGG	3.55E-05	0.135	CAMP phosphodiesterases class-II:metallo-beta-lactamase superfamily	Beta-lactamase superfamily domain-containing protein
scaffold23163_6.4_0.605_8521_		ACCTATCACACCGAGGAGCT	5.44E-05	0.135	N.A.	acetyl-CoA carboxylase carboxyl transferase subunit beta; accD
scaffold80788_6.6_0.617_8275_		TCCTAC ACCATATGGCAGTC	3.64E-05	0.0438	N.A.	mannosyl-3-phosphoglycerate phosphatase
scaffold80788_6.6_0.617_8275_		GACTGCCATATGGTGTAGGA	4.02E-05	0.0438	N.A.	Hypothetical protein
scaffold158494_8.8_0.642_6111_	Nspbox 51	CCCTGCCGACTGTCGCAGGC	4.74E-05	0.0477	Outer membrane protein A precursor	OmpA-OmpF porin, OOP family
scaffold158494_8.8_0.642_6111_	Nspbox 52	GCCTGCGACAGTCGGCAGGG	5.16E-05	0.0477	IMP cyclohydrolase (EC 3.5.4.10) /	phosphoribosylaminoimidazolecarboxamide

					Phosphoribosylaminoimidazolecarboxamide formyltransferase (EC 2.1.2.3)	formyltransferase / IMP cyclohydrolase; purH
scaffold31827_6.0_0.595_6201_		AACTGGAAAGACGGCCAGGA	3.33E-05	0.0511	N.A.	Hypothetical protein
scaffold3684_5.4_0.615_6830_		TCCTGCTCGATCAGATAGCT	4.80E-06	0.011	N.A.	Uncharacterized conserved protein GlcG, DUF336 family
scaffold3684_5.4_0.615_6830_		CCCTACCGGACCTCTCAGGA	3.40E-05	0.0391	N.A.	3-hydroxyisobutyrate dehydrogenase (EC 1.1.1.31)
scaffold66968_5.8_0.600_6516_		AGCAGCCAGGTCGGGCAGGG	1.46E-05	0.0388	N.A.	glycyl-tRNA synthetase beta chain (EC 6.1.1.14)
scaffold26825_5.7_0.596_7798_	Nspbox 53	CCCTGCCTGATGCGTCAGGA	1.43E-05	0.0656	NAD(P)H dehydrogenase (quinone)	Putative NADPH-quinone reductase (modulator of drug activity B)
scaffold170536_6.3_0.597_8634_		AGCAGTCCGGTCGTGCAGGA	4.05E-05	0.115	N.A.	tryptophanyl-tRNA synthetase (EC 6.1.1.2); trpS
scaffold7091_6.5_0.621_15153_		ACCTGTTCAACCTGGAAGAT	5.38E-05	0.188	N.A.	3-hydroxyisobutyrate dehydrogenase
scaffold46559_5.9_0.609_5748_		CCCTCCCCCACTGACAGTG	6.87E-05	0.0778	N.A.	hydroxymethylpyrimidine/phosphomethylpyrimidine kinase; thiD
scaffold66986_6.0_0.595_6903_		GTCTACCCTCTCTGGCAGGC	9.99E-05	0.327	N.A.	indoleamine 2,3-dioxygenase
scaffold93802_5.1_0.579_6536_		ACCTGGCGGCACTGGCAGTC	6.88E-06	0.0238	N.A.	Predicted thiol-disulfide oxidoreductase YuxK, DCC family

scaffold102058_5.2_0.594_4190_		ACCTGGCCAAGGTGGTAGG	1.25E-05	0.0109	N.A.	
scaffold151239_5.5_0.591_4961_	Nspbox 54	GCCTGTAGGAACAAGCAGTT	1.77E-05	0.0334	NADP-dependent malic enzyme	malate dehydrogenase (oxaloacetate-decarboxylating)
scaffold33413_5.2_0.602_4892_	Nspbox 55	CCCTGTCCGATTCGGCAGGA	1.78E-07	0.000546	NAD(P)H dehydrogenase (quinone)	Putative NADPH-quinone reductase (modulator of drug activity B)
scaffold142406_5.7_0.613_5210_	Nspbox 56	AACTGCCGGATCTGGAAGAA	4.44E-05	0.102	Soluble pyridine nucleotide transhydrogenase (EC 1.6.1.1)	NAD(P) transhydrogenase; sthA
scaffold47357_7.0_0.604_5508_		ACCTATCAGGTCTTGCTGGA	8.83E-06	0.0271	N.A.	Hypothetical protein
scaffold162630_11.6_0.594_8081_	NA	CCCTGTCCGCTAGGGCTGGG	5.28E-05	0.966	Glycoprotein gp2	N.A.
scaffold25998_5.1_0.613_7658_		GGCTGCCCGATGCGAGAGGG	8.85E-05	0.222	N.A.	CBS domain-containing protein
scaffold92900_6.7_0.587_9260_		TCCTGTACGCATTGGCAGGC	4.83E-06	0.00909	N.A.	starch phosphorylase
scaffold94049_6.7_0.615_8738_		AGCTGCCTGCTCGAACAGGC	1.22E-05	0.0421	N.A.	uridylate kinase (EC 2.7.4.22); pyrH
scaffold112779_5.8_0.603_3447_		TCCTGCTTGATGATGCAGGC	5.35E-05	0.0757	N.A.	trans-2,3-dihydro-3-hydroxyanthranilate isomerase
scaffold118398_6.9_0.632_3360_		ACCAGCACGATGGTACAGGC	3.36E-05	0.038	N.A.	haloacid dehalogenase superfamily, subfamily IA, variant 3 with third motif having DD or ED
scaffold158167_5.0_0.601_3851_		TCCTACCAGGTTTTGCTGGA	6.72E-05	0.154	N.A.	Hypothetical protein

scaffold168646_5.8_0.572_3973_	Nspbox 57	ACCTACCGAATCTTGCTGGA	1.66E-05	0.0255	ATP synthase beta chain	ATP synthase F1 subcomplex beta subunit
scaffold70961_4.6_0.590_3250_	Nspbox 58	ACCTGTATCATCCGGTTGGG	8.66E-05	0.112	Universal stress protein family	Nucleotide-binding universal stress protein, UspA family
scaffold94286_6.3_0.598_3567_		TCCTGCCCGTTACAGCAGGC	2.86E-05	0.0324	N.A.	sulfate transport system substrate-binding protein
C14184373_11.0_0.563_4545_		GGCTACCATATCGGAGAGGG	7.32E-05	0.11	N.A.	purine-binding chemotaxis protein CheW
scaffold146407_9.0_0.618_8472_	Nspbox 59	ACCTATTCGTTCCGGCAGCA	1.18E-05	0.0273	Methionine ABC transporter ATP-binding protein	putative ABC transport system ATP-binding protein; ybbA
scaffold165391_5.4_0.595_4270_		AACAATACGTTCGTGCAGG	1.67E-05	0.0257	N.A.	Hypothetical protein
scaffold174800_5.5_0.596_3043_		CCCTCGCCGATTTGCGAGGT	5.41E-05	0.0568	N.A.	Chemoreceptor zinc-binding domain-containing protein
scaffold174800_5.5_0.596_3043_		ACCTCGCAAATCGGCGAGGG	6.16E-05	0.0568	N.A.	Predicted arabinose efflux permease, MFS family; NIDE1691
scaffold18118_4.5_0.578_2602_		ACCTGCTGGTTTCGGCAGAG	4.17E-05	0.0252	N.A.	Hypothetical protein
scaffold18118_4.5_0.578_2602_		CTCTGCCGAAACCAGCAGGT	4.41E-05	0.0252	N.A.	Lon protease (S16) C-terminal proteolytic domain-containing protein
scaffold36806_5.0_0.610_2469_		ACCTATCACACCGAGGAGCT	5.44E-05	0.0185	N.A.	acetyl-CoA carboxylase carboxyl transferase subunit beta; accD

scaffold49794_6.1_0.577_2331_	Nspbox 60	CGCTGCAATAACTCATAGGG	7.15E-05	0.0778	Flagellar biosynthesis protein FliS	flagellar protein FliS
scaffold67438_5.5_0.601_2521_		ATCTGCAAGTCGGTGCAGGG	3.36E-05	0.0126	N.A.	branched chain amino acid aminotransferase apoenzyme (EC 2.6.1.42)
scaffold79922_6.6_0.610_2155_	Nspbox 61	CCCTAGCCAATGGCTCAGGT	6.64E-05	0.039	Regulator of competence-specific genes	DNA transformation protein
scaffold79922_6.6_0.610_2155_		ACCTGAGCCATTGGCTAGGG	6.79E-05	0.039	N.A.	hypothetical protein
scaffold81191_5.2_0.629_2595_	Nspbox 62	GCCTGCAAGAAGGGATAGCG	4.12E-05	0.0192	oxidoreductase, FAD-binding	Phytoene dehydrogenase-related protein
scaffold81191_5.2_0.629_2595_		ACCTTCACCGACCGATAGGT	4.83E-05	0.0192	N.A.	23S rRNA pseudouridine2605 synthase
scaffold81191_5.2_0.629_2595_		ACCTATCGGTCGGTGAAGGT	5.01E-05	0.0192	N.A.	Phytoene dehydrogenase-related protein
scaffold104361_4.7_0.549_4622_		AACTAGAGCACTTGACAGGG	4.83E-05	0.148	N.A.	AraC-type DNA-binding protein
scaffold134837_7.9_0.572_3900_		ACCTCTCCGGCCTCGCAGGC	2.00E-05	0.0184	N.A.	RHS repeat-associated core domain-containing protein
scaffold134837_7.9_0.572_3900_	Nspbox 63	GCCTGCGAGGCCGGAGAGGT	2.32E-05	0.0184	Transposase	Transposase
scaffold176218_9.0_0.529_4362_		CGCTACCGTTTTGGCCAGGT	5.44E-05	0.1	N.A.	Transposase
C13989851_26.0_0.614_1673_	Nspbox 64	GCCTATACCATCTGCCAGCG	4.74E-05	0.0364	Ferric siderophore transport system,	outer membrane transport energization

					biopolymer transport protein ExbB	protein ExbB (TC 2.C.1.1.1)
scaffold147101_8.3_0.614_1766_	Nspbox 65	TCCTGACGGCACGAACAGGG	5.81E-05	0.0437	putative inner membrane protein	ATP:ADP antiporter, AAA family
scaffold3761_5.8_0.618_1934_		ACCTGCATCCGCTGACAGTG	1.14E-05	0.00524	N.A.	hydroxymethylpyrimidine/phosphomethylpyrimidine kinase
scaffold25247_5.6_0.596_2739_		ACCTGGCGGCATTGGCAGTC	1.45E-05	0.0213	N.A.	Predicted thiol-disulfide oxidoreductase YuxK, DCC family
scaffold70435_7.5_0.612_62683_		GCCTACCCGCTCAAACAGCT	2.71E-05	0.51	hypothetical protein	hypothetical protein
scaffold47404_7.5_0.611_59229_	Nspbox71	ACCTGACGGAGCAGTCAGGT	2.96E-06	0.0619	hypothetical protein	hypothetical protein (Conserved exported protein)
scaffold142530_8.4_0.614_43758_		ACCTACGATCCCCTGCAGGC	6.72E-05	0.995	hypothetical protein	hypothetical protein
scaffold159073_8.0_0.602_27142_		GCCTATCAATCCGGGCAGTT	3.38E-06	0.0311	N.A.	Nucleoside phosphorylase
scaffold22133_7.1_0.610_30015_		CCCTGCAGGCTCCAGCAGGA	1.32E-05	0.157	N.A.	protein of unknown function (DUF4124)
scaffold91289_7.5_0.608_26333_		TCCTGCAGGACGTACCAGGT	2.49E-05	0.241	DNA polymerase beta domain protein regio	Nucleotidyltransferase domain-containing protein
scaffold25542_6.0_0.585_14364_		ACCTGCCAACGCTCCAGGC	3.49E-06	0.02	N.A.	Cytochrome C oxidase, cbb3-type, subunit III
scaffold121816_7.8_0.600_16717_		ACCTACCTCGGTTCGCAGGG	5.88E-05	0.426	N.A.	Nucleotidyltransferase domain-containing protein
scaffold122042_8.5_0.629_42142_		GCCTACCATTATCTGCAGGT	1.97E-05	0.3	hypothetical protein	Putative MetA-pathway of phenol degradation

scaffold2120_6.8_0.612_14221_		GCCTGGCCACCCAGGCAGGG	3.44E-05	0.185	N.A.	negative regulator of flagellin synthesis FlgM
scaffold159574_7.6_0.600_9394_		CCCTGGCGGTTTGGCCAGGA	1.97E-05	0.106	hypothetical protein	hypothetical protein
scaffold18398_7.3_0.591_10742_		CCCTATCATCCCTGCCAGGC	3.48E-05	0.0935	hypothetical protein	hypothetical protein
scaffold81919_5.3_0.625_8803_		GCCTGTACGGTGACACAGGA	8.33E-05	0.256	Uncharacterized iron-regulated membrane protein	hypothetical protein
scaffold43290_6.0_0.603_5925_		ACCTGGAATTCCCGACAGCC	8.42E-05	0.135	hypothetical protein	hypothetical protein
scaffold5089_4.9_0.610_5399_		ACCTACCAAAAGTTGCAGGT	1.90E-07	0.000577	hypothetical protein	hypothetical protein
scaffold12347_5.6_0.603_3877_		GCCTCTTCGTGTTGACAGGT	3.64E-05	0.0333	hypothetical protein	hypothetical protein
scaffold12347_5.6_0.603_3877_		AACTGCAGCTCCGGATAGGC	4.36E-05	0.0333	hypothetical protein	Predicted membrane protein (DUF2127)
scaffold152353_6.2_0.599_3663_		GCCTGGGCGATCTATCAGGT	2.40E-05	0.0294	hypothetical protein	Putative signal transducing protein
scaffold152353_6.2_0.599_3663_		ACCTGATAGATCGCCCAGGC	2.43E-05	0.0294	N.A.	Iron-containing redox enzyme
scaffold174649_6.2_0.605_3293_		GCCTATCCGGAGTTACAGTT	1.64E-05	0.0238	N.A.	hypothetical protein
scaffold174649_6.2_0.605_3293_		AACTGTAACTCCGGATAGGC	1.78E-05	0.0238	N.A.	Uncharacterized membrane protein, DUF2068 family
scaffold38166_4.7_0.599_3585_	Nspbox72	G CCTACCCCGATGGGTAGGT	2.30E-05	0.0175	hypothetical protein	hypothetical protein
scaffold66627_5.8_0.588_3823_		AGCTATCAGGTCTGGCATGT	7.66E-05	0.035	N.A.	Protein of unknown function (DUF3422)

scaffold66627_5.8_0.588_3823_		AGCTACAAATTTCGCGAGGT	9.00E-05	0.035	N.A.	PilZ domain-containing protein
scaffold8938_6.9_0.596_3770_		ACCTATCCGGTCGAGGAGAT	4.30E-05	0.0443	hypothetical protein	hypothetical protein
scaffold164460_8.0_0.549_6825_		CACTGGAACGCCTCGCAGGT	5.91E-05	0.159	hypothetical protein	hypothetical protein
scaffold101376_5.3_0.600_2659_		GCCTGCCCTTTCACGCAGTG	7.11E-05	0.0817	hypothetical protein	Uncharacterized conserved protein YidB, DUF937 family
scaffold109483_4.8_0.596_2391_		CACTGTCATAAGGGGCAGCA	7.61E-05	0.0873	N.A.	hypothetical protein
scaffold149742_5.4_0.598_2687_		ACCTGGACGATGCGCCAGGC	1.35E-05	0.0103	hypothetical protein	hypothetical protein
scaffold149742_5.4_0.598_2687_		TACTGCAAAGTTGTCCAGTG	6.68E-05	0.0255	Opacity protein	hypothetical protein
scaffold22389_4.2_0.615_2323_		TACTGCCTCACCTGGCAGAT	3.44E-05	0.00244	hypothetical protein	hypothetical protein
scaffold37519_7.9_0.575_2583_		GCCTATACCATCGCGCAGGA	2.86E-05	0.0328	hypothetical protein	stage II sporulation protein AA (anti-sigma F factor antagonist)
scaffold53997_4.8_0.595_2198_		CACTCCCGAATATGACAGTT	2.58E-05	0.018	hypothetical protein	hypothetical protein
scaffold53997_4.8_0.595_2198_		ACCAGGATGCTCGGACAGGG	6.12E-05	0.0214	hypothetical protein	hypothetical protein
C13859426_5.0_0.573_1260_		ACCTGGATCTCCAGGTAGGG	8.52E-05	0.0322	hypothetical protein	hypothetical protein
C13922126_60.0_0.586_1426_		ATCTGCAAGGTCTGTCAGCA	3.60E-05	0.0355	hypothetical protein	hypothetical protein
C14020669_5.0_0.596_1824_		TACTGAATGATCCGGCAGGG	1.34E-05	0.0102	hypothetical protein	hypothetical protein
scaffold139276_4.7_0.590_1960_		ACCTGGCACATACTGCAGGC	1.13E-05	0.00433	hypothetical protein	hypothetical protein

scaffold52940_6.7_0.574_2951_		ACCTCCGAGAGCTTCCAGCG	8.42E-05	0.0645	hypothetical protein	Predicted DNA-binding protein, contains XRE-type HTH domain
scaffold90473_3.4_0.594_1040_		CCCTGCATGCGGGTACAGTA	9.67E-05	0.0736	hypothetical protein	hypothetical protein

Supplementary Table 5. Alignment of conserved amino acid residue of the putative *luxIR*

	LuxI	R 25	F 29	W 35	D 46	D 49	R 70	E 101	R 104
	US1	R	F	W	D	D	R	E	R
	US3	R	F	W	D	D	R	E	R
	US4	R	F	W	D	D	R	E	R
	US5	R	F	W	D	D	R	E	R
	US6	R	F	W	D	D	R	E	R
	US7	R	F	W	D	D	R	E	R
	US8	R	F	W	D	D	R	E	R
	US9	R	F	W	D	D	R	E	R
	US11	R	F	**Y**	D	D	R	E	R
	US13	R	**Y**	W	D	D	R	E	R
	US14	R	F	W	D	D	R	E	R
	US15	R	F	W	D	D	R	E	R
	US16	R	F	**D**	D	D	R	E	R

		Acyl-binding site					DNA-binding site		
	**LuxR**	W 66	D 79	P 80	W 94	G 121	E 187	L 191	G 197
**Paired LuxIR**	UR3	W	D	P	W	G	E	L	G
	UR4	W	D	P	W	G	E	**V**	G
	UR9	W	D	P	W	G	E	L	G
	UR11	W	D	P	W	G	**Q**	L	G
	UR24	W	D	P	W	G	E	L	G
	UR29	W	D	P	W	G	E	L	G
	UR43	W	D	P	W	G	E	L	G
	UR45	W	D	P	W	G	E	L	G
	UR52	W	D	P	W	G	E	L	G
**Solo LuxR**	**PluR**	**T**	D	**Q**	**C**	**S**	E	**I**	G
	**PauR**	**T**	D	**Q**	**Y**	**I**	E	**I**	G
	UR5	**F**	D	P	**S**	**C**	E	L	G
	UR6	**F**	D	P	W	G	E	L	G
	UR8	W	D	P	W	**I**	E	L	G
	UR20	**Y**	D	P	W	G	E	L	G
	UR21	**Y**	D	P	**Y**	G	E	L	G

	UR22	**L**	D	**E**	W	**R**	E	L	G
	UR25	**F**	D	P	W	G	E	**T**	G
	UR27	**Y**	D	P	W	G	E	L	G
	UR28	**Y**	**A**	P	W	G	**Q**	L	G
	UR33	**F**	D	P	W	G	E	L	G
	UR34	**Y**	D	P	W	G	E	L	G
	UR35	**Y**	D	P	W	G	E	L	G
	UR40	**L**	D	P	**F**	G	E	**M**	G
	UR41	**Y**	D	P	W	G	E	L	G
	UR42	W	D	**A**	**F**	G	**Q**	L	G
	UR44	**F**	D	P	**S**	**C**	E	L	G
	UR47	**Y**	D	P	**Y**	G	E	L	G
	UR53	W	D	P	**S**	**A**	**D**	L	G
	UR2	W	D	P	W	G	**Q**	L	G
	UR16	W	D	P	W	G	E	L	G
	UR18	W	D	P	W	G	E	L	G
	UR26	W	D	P	W	G	E	L	G

Supplementary Table 6. Regulation of NspR1 on the predicted lux-box containing promoters with and without the co-expression of Nitrospira sigma factors

Nspbox	Fold Change														
	NspR1			NspR1 + RpoD			NspR1 + RpoD2			NspR1 + RpoN			NspR1 + RpoS		
Empty	1.28	±	0.2	1.04	±	0.2	1.01	±	0.2	1.04	±	0.2	1.13	±	0.2
Nspbox1	196.66	±	22.7	7.25	±	10.8	61.34	±	33.9	6.20	±	2.4	137.22	±	67.7
Nspbox2	14.01	±	2.1	4.30	±	2.3	6.02	±	2.6	1.03	±	0.3	5.67	±	3.3
Nspbox3	0.91	±	0.0	0.95	±	0.2	1.21	±	0.1	1.06	±	0.4	1.08	±	0.2
Nspbox4	0.91	±	0.0	0.96	±	0.1	1.22	±	0.2	1.07	±	0.2	1.15	±	0.1
Nspbox5.1	0.91	±	0.0	1.04	±	0.1	1.21	±	0.2	1.00	±	0.1	1.07	±	0.1
Nspbox6	0.92	±	0.1	1.05	±	0.3	1.22	±	0.3	0.99	±	0.2	0.82	±	0.1
Nspbox7	1.02	±	0.1	0.95	±	0.1	1.05	±	0.2	0.99	±	0.4	0.90	±	0.1
Nspbox8	0.92	±	0.1	0.99	±	0.2	1.00	±	0.4	1.17	±	0.1	1.00	±	0.1
Nspbox9	0.95	±	0.0	1.01	±	0.2	1.00	±	0.4	1.17	±	0.1	0.96	±	0.2
Nspbox10	1.00	±	0.0	0.98	±	0.2	0.92	±	0.2	0.96	±	0.3	1.03	±	0.4
Nspbox11	1.01	±	0.1	0.94	±	0.2	1.07	±	0.4	1.09	±	0.2	1.00	±	0.3
Nspbox12	0.98	±	0.0	1.06	±	0.4	1.04	±	0.4	1.11	±	0.1	0.93	±	0.0
Nspbox13	0.94	±	0.0	1.11	±	0.4	0.97	±	0.2	1.10	±	0.1	1.06	±	0.2
Nspbox14	1.02	±	0.0	0.98	±	0.2	1.08	±	0.1	0.96	±	0.0	1.14	±	0.2
Nspbox15	1.00	±	0.0	1.01	±	0.2	1.15	±	0.3	0.92	±	0.1	1.19	±	0.3
Nspbox17	0.79	±	0.3	1.00	±	0.3	1.06	±	0.1	1.09	±	0.3	1.27	±	0.2
Nspbox18	0.88	±	0.1	1.01	±	0.2	1.13	±	0.0	1.08	±	0.1	1.12	±	0.0
Nspbox20	0.78	±	0.0	1.00	±	0.1	1.07	±	0.2	0.97	±	0.1	1.09	±	0.2
Nspbox21	0.81	±	0.0	1.09	±	0.2	1.03	±	0.1	0.97	±	0.2	1.05	±	0.1
Nspbox22	0.96	±	0.0	0.98	±	0.2	1.03	±	0.1	1.27	±	0.4	1.13	±	0.1
Nspbox23	0.99	±	0.1	1.02	±	0.1	1.90	±	0.1	0.98	±	0.2	1.17	±	0.2
Nspbox24	0.89	±	0.0	1.01	±	0.0	0.86	±	0.0	1.03	±	0.1	1.03	±	0.1

Nspbox25	0.87	±	0.0	1.01	±	0.3	0.82	±	0.1	1.15	±	0.4	0.88	±	0.1
Nspbox26	0.87	±	0.2	1.18	±	0.4	1.03	±	0.3	1.15	±	0.2	1.08	±	0.1
Nspbox27	1.00	±	0.0	1.05	±	0.4	1.19	±	0.3	1.31	±	0.6	1.16	±	0.4
Nspbox28	0.96	±	0.0	0.97	±	0.2		*NIL*		0.94	±	0.1	1.07	±	0.1
Nspbox29	0.94	±	0.0	0.99	±	0.3	0.93	±	0.1	1.13	±	0.2	1.06	±	0.1
Nspbox30	0.96	±	0.0	1.02	±	0.2		*NIL*		1.25	±	0.3	1.13	±	0.2
Nspbox31	1.03	±	0.1	1.04	±	0.2	0.91	±	0.1	1.12	±	0.4	0.91	±	0.1
Nspbox32	0.95	±	0.1	1.11	±	0.3	0.91	±	0.1	1.16	±	0.2	1.32	±	0.2
Nspbox34	0.86	±	0.0	0.96	±	0.2	0.94	±	0.1	0.99	±	0.1	0.99	±	0.1
Nspbox35	0.84	±	0.1	1.08	±	0.4	0.76	±	0.2	1.12	±	0.0	0.91	±	0.1
Nspbox36	1.05	±	0.1	0.98	±	0.1	1.14	±	0.0	1.05	±	0.1	1.09	±	0.2
Nspbox37	1.04	±	0.0	0.96	±	0.1	1.13	±	0.0	0.97	±	0.1	1.09	±	0.1
Nspbox38	1.05	±	0.1	0.93	±	0.3	0.89	±	0.1	0.81	±	0.1	1.06	±	0.3
Nspbox40	1.00	±	0.0	1.02	±	0.2	1.07	±	0.3	0.79	±	0.3	0.91	±	0.4
Nspbox41	1.04	±	0.0	0.96	±	0.4	1.18	±	0.4	0.94	±	0.4	1.16	±	0.3
Nspbox42	0.85	±	0.2	0.92	±	0.0	1.12	±	0.3	1.07	±	0.1	0.97	±	0.1
Nspbox44	0.98	±	0.0	1.06	±	0.2	0.87	±	0.2	0.89	±	0.3	1.00	±	0.2
Nspbox45	0.94	±	0.1	0.81	±	0.1		*NIL*		1.03	±	0.1	1.03	±	0.2
Nspbox46	1.03	±	0.1	1.03	±	0.2	1.11	±	0.3	0.95	±	0.1	0.91	±	0.2
Nspbox47	0.99	±	0.0	0.94	±	0.1		*NIL*		1.01	±	0.0	0.88	±	0.1
Nspbox49	1.01	±	0.0	0.99	±	0.3	1.28	±	0.4	0.93	±	0.4	1.22	±	0.2
Nspbox50	0.99	±	0.0	0.81	±	0.2	1.03	±	0.1	0.83	±	0.1	1.03	±	0.1
Nspbox51	1.01	±	0.0	0.81	±	0.2	1.04	±	0.1	0.69	±	0.1	1.03	±	0.1
Nspbox52	0.96	±	0.1	0.94	±	0.3	1.22	±	0.1	0.85	±	0.1	1.03	±	0.1
Nspbox53	1.02	±	0.0	1.25	±	0.4	0.80	±	0.1	0.94	±	0.2	1.18	±	0.4
Nspbox54	0.93	±	0.1	0.83	±	0.2	1.33	±	0.3	0.79	±	0.2	1.10	±	0.1
Nspbox55	0.96	±	0.0	0.89	±	0.1	1.30	±	0.3	0.97	±	0.2	1.33	±	0.4
Nspbox56	0.96	±	0.1	0.86	±	0.3	1.30	±	0.3	1.12	±	0.1	1.28	±	0.3

Nspbox58	1.00	±	0.0	0.96	±	0.1	0.97	±	0.1	1.21	±	0.4	1.02	±	0.1
Nspbox59	0.98	±	0.0	0.99	±	0.3	0.93	±	0.2	0.95	±	0.2	0.94	±	0.0
Nspbox60	1.04	±	0.2	1.15	±	0.4	1.00	±	0.1	1.26	±	0.6	0.98	±	0.1
Nspbox61	0.86	±	0.0	1.17	±	0.5	1.08	±	0.3	1.13	±	0.5	0.94	±	0.2
Nspbox62	0.88	±	0.0	0.78	±	0.1	0.96	±	0.1	0.99	±	0.2	1.12	±	0.2
Nspbox65	0.87	±	0.0	1.09	±	0.2	0.92	±	0.3	1.10	±	0.3	0.95	±	0.1
Nspbox68	1.06	±	0.1	1.14	±	0.1	0.94	±	0.1	1.06	±	0.4	1.11	±	0.3
Nspbox69	1.41	±	0.1	1.01	±	0.4	1.11	±	0.2	1.12	±	0.3	*NIL*		
Nspbox70	1.31	±	0.1	0.89	±	0.3	1.14	±	0.1	1.06	±	0.4	0.91	±	0.2
Nspbox71	1.20	±	0.0	0.80	±	0.3	1.14	±	0.1	*NIL*			1.15	±	0.1
Nspbox72	1.32	±	0.0	0.95	±	0.1	1.08	±	0.1	*NIL*			1.04	±	0.2

Screening was performed in E. coli TOP10 transformed with pPROBE-gfp(ASV) containing the predicted lux-box containing promoters (Nspbox) and pUCP22-NspR1 or pUCP22-NspR1 carrying the Nitrospira sigma factor gene.. Relative fold induction were calculated by measuring the fold induction of GFP signals of induced sample (added with 1 mM C8-HSL) to uninduced control (added with DMSO), and normalized to fold induction of GFP from the empty pPROBE-gfp(ASV) vector control. Data are presented as mean ± SD.

Supplementary Figures

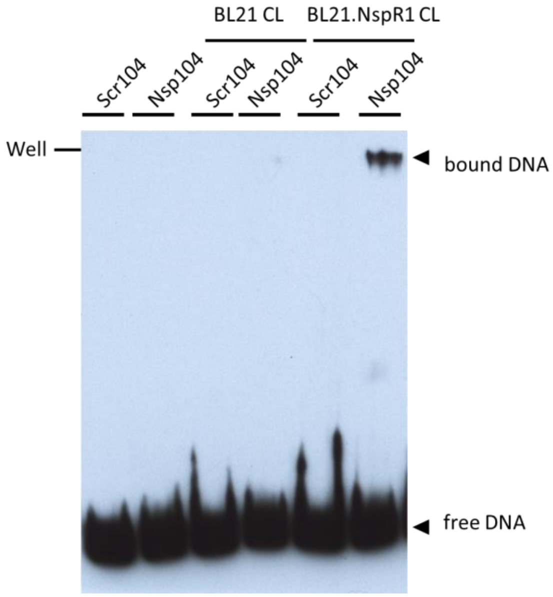

Supplementary Figure 1. Addition of cell lysate from NspR1-expressing BL21 strain induced band shift in Nsp104 but not Scr104. Solid arrows indicate bound DNA and free DNA.

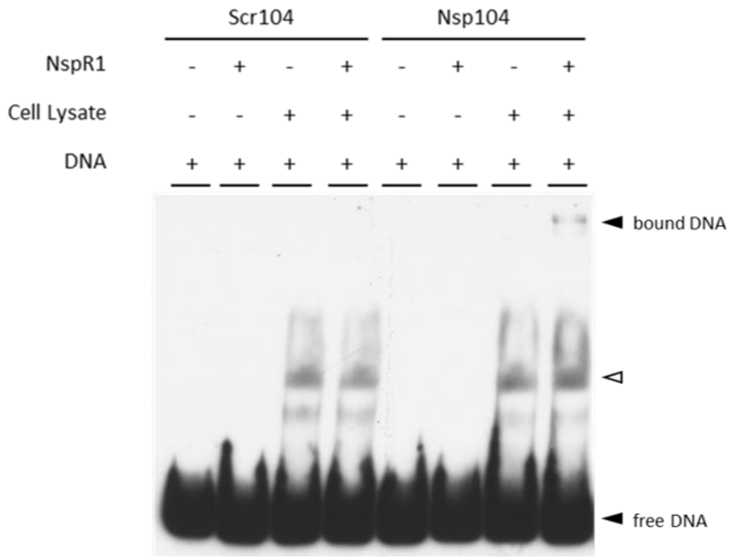

Supplementary Figure 2. Supplementing BL21 cell lysate with purified NspR1 induced band shift in Nsp104 but not Scr104. Solid arrows indicate bound DNA and free DNA while open arrow indicate non-specific DNA-binding of cell lysate origin.

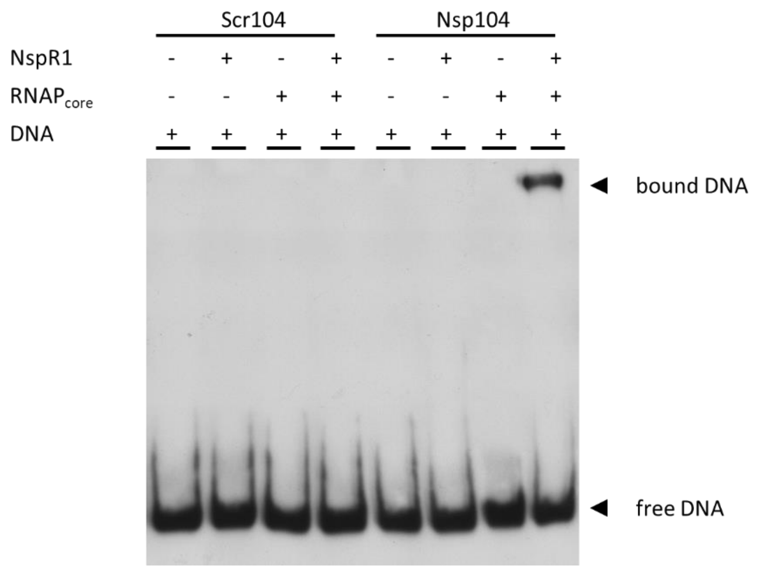

Supplementary Figure 3. Co-binding of purified NspR1 with *E. coli* RNAP$_{core}$ to Nsp104.

Solid arrows indicated bound DNA and free DNA.

Raw images of blots/gels

Figure 5a

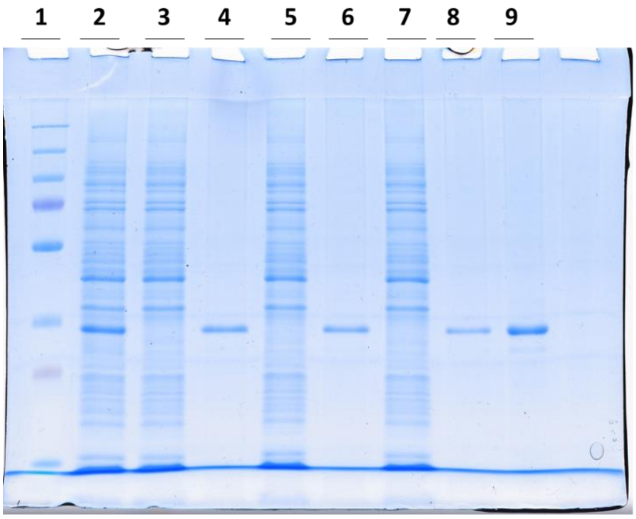

Samples

1. PageRuler Plus ladder
2. BL21.NspR1 cell lysate
3. Flow-through (replicate#1)
4. Elution (replicate#1)
5. Flow-through (replicate#2)
6. Elution (replicate#2)
7. Flow-through (replicate#3)
8. Elution (replicate#3)
9. concentrated NspR1

Figure 5b

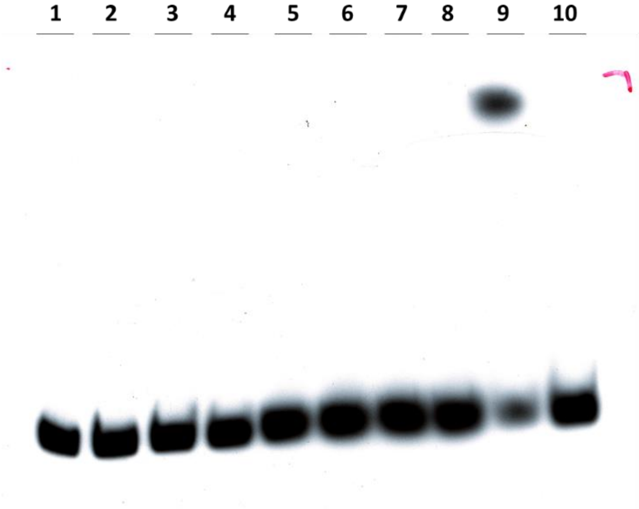

Samples

1. Scr104
2. Scr104 + NspR1
3. Scr104 + RNAP$_{holo}$
4. Scr104 + RNAP$_{holo}$ + NspR1
5. Scr104 + NspR1 + BL21 cell lysate
6. Nsp104
7. Nsp104 + NspR1
8. Nsp104 + RNAP$_{holo}$
9. Nsp104 + RNAP$_{holo}$ + BL21 cell lysate

Supplementary Figure 1

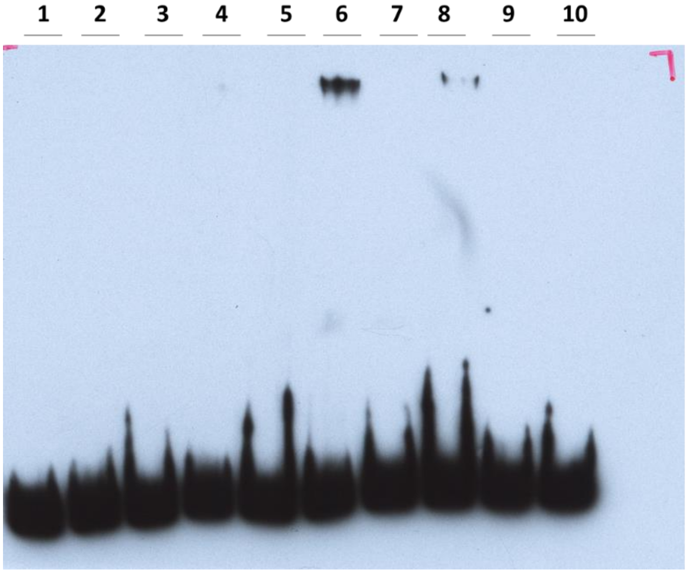

Samples

1. Scr104
2. Nsp104
3. Scr104 + BL21 cell lysate
4. Nsp104 + BL21 cell lysate
5. Scr104 + BL21.NspR1 cell lysate (0.1 mM IPTG)
6. Nsp104 + BL21.NspR1 cell lysate (0.1 mM IPTG)
7. Scr104 + BL21.NspR1 cell lysate (uninduced)
8. Nsp104 + BL21.NspR1 cell lysate (uninduced)
9. Scr104 + BL21.NspR1 cell lysate (1 mM IPTG)
10. Nsp104 + BL21.NspR1 cell lysate (1 mM IPTG)

Supplementary Figure 2

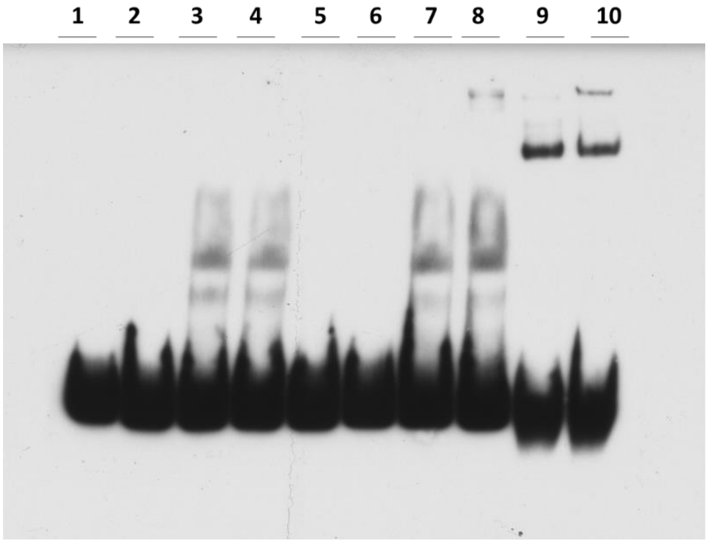

Samples

1. Scr104
2. Scr104 + NspR1
3. Scr104 + BL21 cell lysate
4. Scr104 + BL21 cell lysate + NspR1
5. Nsp104
6. Nsp104 + NspR1
7. Nsp104 + BL21 cell lysate
8. Nsp104 + BL21 cell lysate + NspR1
9. SELEX_R0 + RNAPcore
10. SELES_R0 + RNAPcore + NspR1

Supplementary Figure 3

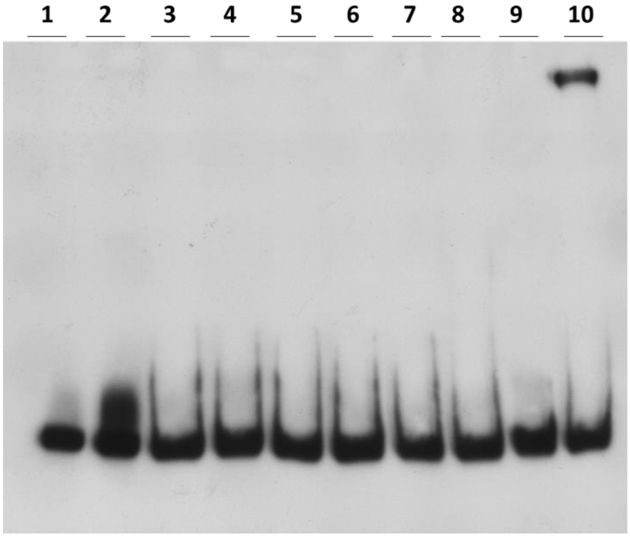

Samples

1. SELEX_R2 + NspR1
2. SELEX_R2 + NspR1 + $RNAP_{core}$
3. Scr104 + NspR1
4. Scr104 + $RNAP_{core}$
5. Scr104 + NspR1 + $RNAP_{core}$
6. Nsp104
7. Nsp104 + NspR1
8. Nsp104 + $RNAP_{core}$
9. Nsp104 + NspR1 + $RNAP_{core}$
